# BOBA FRET: Bootstrap-Based Analysis of Single-Molecule FRET Data

**DOI:** 10.1371/journal.pone.0084157

**Published:** 2013-12-27

**Authors:** Sebastian L. B. König, Mélodie Hadzic, Erica Fiorini, Richard Börner, Danny Kowerko, Wolf U. Blanckenhorn, Roland K. O. Sigel

**Affiliations:** 1 Institute of Inorganic Chemistry, University of Zurich, Zurich, Switzerland; 2 Evolutionary Biology and Environmental Studies, University of Zurich, Zurich, Switzerland; University of Alberta, Canada

## Abstract

Time-binned single-molecule Förster resonance energy transfer (smFRET) experiments with surface-tethered nucleic acids or proteins permit to follow folding and catalysis of single molecules in real-time. Due to the intrinsically low signal-to-noise ratio (SNR) in smFRET time traces, research over the past years has focused on the development of new methods to extract discrete states (conformations) from noisy data. However, limited observation time typically leads to pronounced cross-sample variability, i.e., single molecules display differences in the relative population of states and the corresponding conversion rates. Quantification of cross-sample variability is necessary to perform statistical testing in order to assess whether changes observed in response to an experimental parameter (metal ion concentration, the presence of a ligand, etc.) are significant. However, such hypothesis testing has been disregarded to date, precluding robust biological interpretation. Here, we address this problem by a bootstrap-based approach to estimate the experimental variability. Simulated time traces are presented to assess the robustness of the algorithm in conjunction with approaches commonly used in thermodynamic and kinetic analysis of time-binned smFRET data. Furthermore, a pair of functionally important sequences derived from the self-cleaving group II intron *Sc.ai5γ* (d3'EBS1*/IBS1*) is used as a model system. Through statistical hypothesis testing, divalent metal ions are shown to have a statistically significant effect on both thermodynamic and kinetic aspects of their interaction. The Matlab source code used for analysis (bootstrap-based analysis of smFRET data, BOBA FRET), as well as a graphical user interface, is available via http://www.aci.uzh.ch/rna/.

## Introduction

Förster Resonance Energy Transfer (FRET), distance-dependent energy transfer via a long-range dipole-dipole interaction, occurs between a donor fluorophore and an acceptor, which is typically (but not necessarily) also a fluorophore [1]. FRET results in a decrease in both donor emission intensity and lifetime, as well as the appearance of acceptor fluorescence [2]. Monitoring FRET between a single pair of dyes (smFRET) attached to a biomolecule can resolve both static and dynamic heterogeneity within a sample, *i.e.* differences between molecules and time-dependent conformational changes of individual molecules, both of which would otherwise be hidden through ensemble averaging [3], [4]. smFRET experiments are performed either on freely diffusing or surface attached molecules, the latter approach allowing for observation over an extended period of time. Technically, experiments with diffusing samples are implemented using a confocal microscope suitable for single-photon detection (time-correlated single photon counting, TCSPC). Experiments involving surface-tethered molecules can also be conducted with the aforementioned confocal microscope setup [5], although a wide-field or total internal reflection geometry is typically used for excitation, followed by detection with a CCD camera, resulting in time-binned FRET trajectories [6], [7]. Statistical analysis of such time-binned data is the objective of this article.

**Figure 1:**
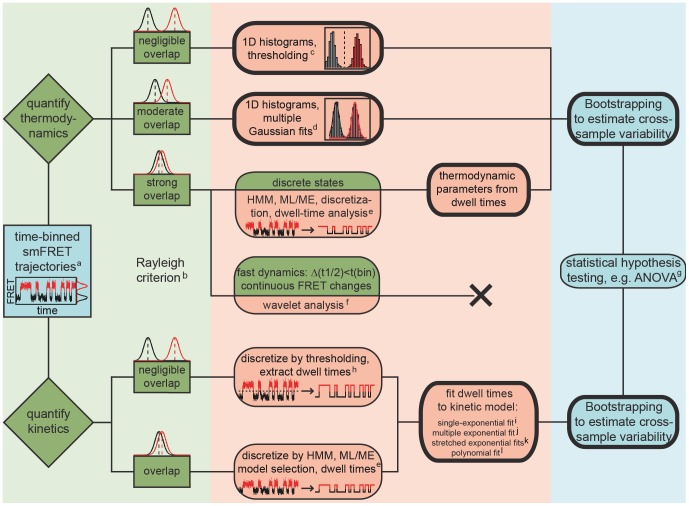
Generalized scheme for analyzing time-binned smFRET data. Bootstrapping can be used both in thermodynamic and kinetic analysis and is compatible with numerous data formats. Bold frames indicate functionalities available in BOBA FRET. ^a)^As defined in the introduction, see also Gopich and Szabo [37]. ^b)^Rayleigh criterion: two subpopulations are indistinguishable when their peak positions are separated by one standard deviation or less [2]. ^c)^See [9]. ^d)^See [10], [34], [35]. ^e)^See [5], [8], [25]–[28], [32]. ^f)^See [18], [19]. ^g)^Multivariate tests (MANOVA) are conceivable to assess whether two or more outcome variables are significantly different at a time, for example the center and the width of a FRET distribution [66]. ^h)^See [12]. i) ^j)^See [34]. ^k)^See [14], [64]. ^l)^Typically used in fluorescence correlation spectroscopy (FCS) [78].

As smFRET data are generated from the emission of single fluorophores, the signal-to-noise ratio (SNR) is generally an issue, and considerable effort has been geared towards the development of tools to analyze noisy time traces. Ideally, such tools should permit to determine the number of conformational states in the system, their relative occurrence, and the rates at which they interconvert [8]. Cumulated FRET histograms have proven useful for simple two- or three-state systems, in which the approximation of individual FRET distributions with a normal distribution leads to minimal discrepancies [2]. When there is no or minimal overlap between the FRET distributions, the relative occurrence of the states is quantified by defining arbitrary cutoff values between FRET distributions (thresholding, Figure 1) [9]. In the case of moderate overlap, multiple Gaussian fits are typically performed to extract quantitative information (Figure 1) [10]. Under these circumstances, dwell times, *i.e.* the time spent in a certain FRET state until a conformational change occurs, can also be easily determined by thresholding, typically followed by fitting the dwell time histograms to exponential decay models to extract the rates of conformational rearrangement (Figure 1) [11]–[14]. However, when the SNR deteriorates (short exposure times or fluorescence quenching) and/or the centers of FRET distributions come close (similar interdye distances or modest conformational dynamics), these straightforward approaches can no longer be sensibly applied (Rayleigh criterion, Figure 1).

Noise in smFRET time traces can be reduced through smoothing, *i.e.* by averaging out the inherent noise of the data collection process and hence emphasizing the discrete nature of the FRET levels [15]. While linear rolling point averaging (also: moving or sliding averaging) is known to obscure transitions with dwell times shorter than the averaging window, the more sophisticated non-linear forward backward filter initially proposed by Chung and Kennedy and adapted by Haran partly overcomes this problem [16], [17]. Nevertheless, it also tends to average out very brief excursions to conformational intermediates in our hands. Taylor *et al.* recently presented an implementation of wavelet shrinkage to denoise smFRET time trajectories (Figure 1) [18], [19]. Here, the observed time series are transformed into a frequency component, followed by suppression of the noise assumed to lie within the high-frequency region of the transformation and inversion of the transformation that yields (in theory) a denoised dataset [18], [20]. It should be noted, however, that noise and signal often overlap in smFRET data, and thus such transformations may lead to spurious oscillations close to the transition (Gibb's phenomenon) [21]. A further application of wavelet transformation is termed change-point identification and has recently been implemented to denoise smFRET data [22]. An extensive overview of strategies for noise removal in so-called piecewise constant signals (constant signal levels connected by abrupt transitions) has been given elsewhere [21].

Hidden-Markov modeling (HMM, Figure 1) was first applied on TCSPC data by Yang and Xie [23], [24], and later utilized for analyzing time-binned FRET trajectories by the groups of Ha (“HaMMy”, [8]), Gonzalez Jr. (“vbFRET”, [25]), Herschlag (“SMART”, [26]), and Dillingham (“CSSR”, [27]), as well as groups from other research fields (“QuB”, [28]). Briefly, a Markov process is a sequence of state-to-state transitions, becoming “hidden” because of the experimental noise [8]. Consequently, HMM attempts to reconstruct the underlying time trace based on transition probabilities of a molecule from a state A to a state B, and emission probabilities, *i.e.* the likelihood of observing a FRET value when the system is in a discrete state *l* assuming the noise can be modeled by a given statistical distribution [10], [29]. Different approaches have been employed to determine the exact number of states: (i) deliberate overfitting followed by model selection using the Bayesian information criterion (BIC) or the Akaike information criterion (AIC) [8], [25], [27], or (ii) a maximum evidence approach for both model selection and determination of the model parameters [25]. Hidden Markov approaches enjoy great popularity nowadays such that an extensive body of literature has been published on this topic, including implementations for short time traces [30], [31] and multivariate HMM dealing with more than one time trace at a time [5], [32]. Nonetheless, it should be mentioned that the basic assumptions do not always hold true for single-molecule processes (single-exponential kinetics, *vide infra*), especially when memory effects or large variations in folding kinetics are observed that go beyond the scope of classical kinetics [18], [33].

With the cumulated histograms and/or the dwell times at hand, both the thermodynamic equilibrium and the kinetics associated with the conformational changes can be characterized. To this end, the corresponding error is typically estimated via the goodness of the fit to the data (GOF) [34], [35]. The GOF reports on how well the model describes the experimental data and is mainly determined by the SNR. Important contributions to the noise are made by the stochastic nature of photon emission (shot-noise), background noise, electron multiplier noise, read-out noise, dark noise, resolution-induced noise [3], [36]–[41], as well as photophysical effects like quantum yield fluctuations and spectral changes or technical aberrations such as focal drift or fluctuations in laser intensity [3], [41], [42]. In turn, this approach neglects cross-sample variability (differences between single molecules) as it relies on building an ensemble from all smFRET time traces at once. Single-molecule data are however known to frequently display intermolecular heterogeneities that may originate from limitations with regard to the observation time (photobleaching) or technical issues. These frequently manifest as pronounced differences regarding the relative population of conformational states, and as differences in the absolute FRET values observed between individual smFRET time traces (heterogeneous broadening) [33], [43]. Consequently, approximation of the error by the GOF is expected to underestimate the variance at the expense of the robustness of data interpretation. It must be emphasized that precise estimation of the variance of the sample is crucial in order to assess whether a difference between different treatment groups is real or has occurred solely by chance, for example a change in the relative population of the conformational states in response to the addition of a small molecule. Such statistical testing has, to the best of our knowledge, not been reported in the field of single-molecule FRET.

Pioneered by Efron [44], the bootstrap scheme is a resampling method to assess the accuracy of sample estimates that has since been applied in numerous branches of biological research including phylogenetics [45], environmental science [46], force-based single-molecule biophysics [47], [48], or molecular dynamics simulations in conjunction with smFRET experiments on freely diffusing molecules [49]. In bootstrapping, the distribution of the whole population, including measures of variance, is estimated from a sample distribution of the size *n* (*n* replicates) [51]. During the resampling process, *N* values of the sample distribution are randomly selected with an equal probability of 1/*N* and multiple selections are allowed (resampling with replacement) [50]. Typically, *N* = n to avoid pseudoreplication and the resampling procedure is repeated *M* times to compute the variance, where 100≤*M*≤500 is usually considered sufficiently robust in phylogenetic research, though more conservative approaches may involve several thousand rounds of bootstrapping [46].

**Figure 2:**
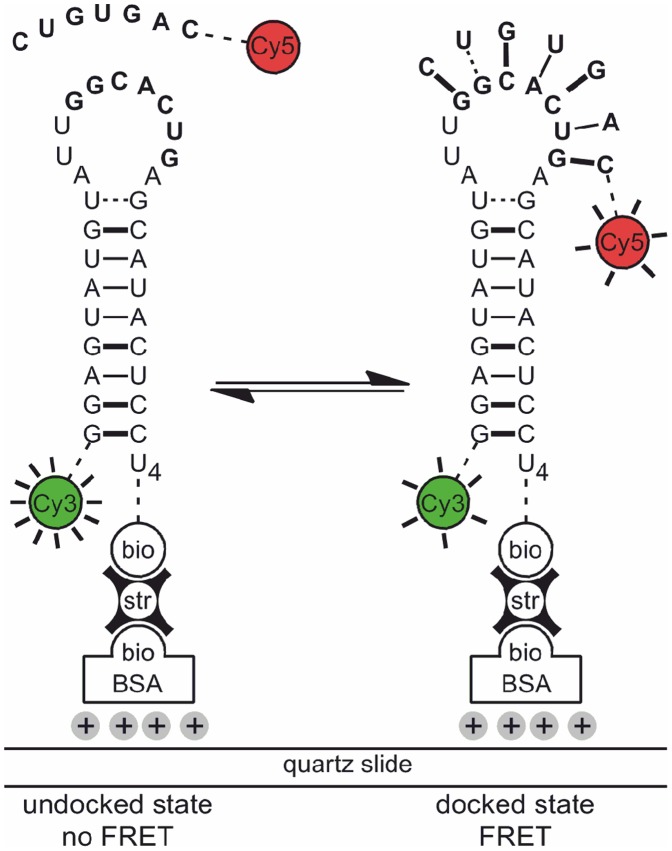
Studying d3'EBS1*/IBS1* interaction by smFRET. The d3'EBS1* hairpin is labeled with Cy3 and tethered to the surface of a quartz slide passivated with biotinylated BSA via a biotin-streptavidin linkage. Docking of a Cy5-IBS1* strand is characterized by the appearance of Cy5 fluorescence and a decrease in Cy3 emission due to FRET. Figure adapted from [70].

To meet the challenge of making smFRET data analysis more robust, we have designed a software package called BOBA FRET (BOotstrap-BAsed analysis of smFRET data) to estimate the cross-sample variability associated with time-binned smFRET measurements using Efron's bootstrap (Figure 1) [44]. The program is freely available and its implementation is straightforward. Herein, we illustrate its workflow to perform both thermodynamic and kinetic analysis of smFRET data: First, the algorithm is shown to be compatible with well-established approaches to analyze smFRET time traces and characterize its robustness using a set of simulated data. Second, BOBA FRET is applied to an experimental dataset, the cation-dependent interaction of the exon-binding sequence 1 (d3'EBS1*) and the intron-binding sequence 1 (IBS1*), which are derived from a crucial part of the 5′splice site recognition complex in the group II intron *Sc.ai5γ* found in *Saccharomyces cerevisiae* (Figure 2). With the bootstrapped errors at hand, we perform statistical hypothesis testing to assess whether cation-induced effects on interaction kinetics and shifts conformational equilibrium are statistically significant [51]–[53].

## Materials and Methods

### Simulations

smFRET time traces were simulated for an intramolecular two-state system. First, discretized time traces were created under the assumption that state-to-state transitions are governed by single-exponential kinetics, followed by addition of Gaussian noise. Standard parameters were based on previous simulations and defined as follows: *FRET*_A_ = 0.3 (undocked state), *FRET*_B_ = 0.7 (docked state); *SNR* = 3.5 (average total intensity = 24.5 photons bin^−1^ s^−1^); SNR distribution width = 0; observation time = 4000 s; *k*_docking_ = 0.1 s^–1^, *k*_undocking_ = 0.04 s^–1^ (average number of transitions = 114 per time trace) [8]. For each set of parameters, 100 time traces were analyzed, followed by an estimation of the cross-sample variability (*vide infra*). All simulations were performed using a home-built script written in MATLAB.

### Oligonucleotides

The RNA sequence pair was derived from the exon-binding site 1 (EBS1) and the intron-binding site 1 (IBS1) found in the primary *cox1* transcript in *cerevisiae*. They are referred to as d3'EBS1* and IBS1* according to the nomenclature used in previous studies (Figure 2) [33], [51]. Labeled oligonucleotides were purchased PAGE-purified from IBA AG (Göttingen, Germany) and additionally HPLC purified [54]. All chemicals were purchased from Sigma-Aldrichs (Buchs, Switzerland).

### smFRET Imaging

Microfluidic channels for total internal reflection microscopy (TIRFM) were prepared from quartz slides (Finkenbeiner, Waltham, MA) as described [55]. The inner surface of the chamber was passivated with biotinylated BSA (Sigma-Aldrich, Buchs, Switzerland), and Cy3-labeled d3'EBS1* was immobilized via a biotin-streptavidin linkage (Figure 2) [56]. The smFRET imaging buffer contained 50 mM MOPS, 100 mM KNO_3_, 1 mM M(NO_3_)_2_ (M^2+^ = Ni^2+^ or Co^2+^), 1% D-glucose, 165 U/mL glucose oxidase, 2170 U/mL catalase, 1 mM Trolox, 25 nM Cy5-labeled IBS1*, pH 6.90 [57]. Cy3 and Cy5 emission levels were monitored in a prism-based total internal reflection fluorescence microscope upon alternating laser excitation (ALEX) as described elsewhere [56], [58]. Briefly, fluorophores were excited at 532 and 640 nm in an alternating fashion using diode lasers (CrystaLaser lc., Reno, NV, USA) attenuated to an intensity of ∼5 mW using neutral density filters (Laser2000 GmbH, Wessling, Germany). Fluorophore emission was spectrally separated with dichroic mirrors (AHF AG, Tübingen, Germany) and projected side-by-side onto a CCD camera (Andor Technology plc., Belfast, Northern Ireland). Photons were collected over 6 minutes at a spatial resolution of 256×256 pixels and a time resolution of 100 ms.

### Data Analysis

smFRET movies were analyzed with a home-built Matlab software (Matlab version 8.20.701, license 49040, MathWorks, Nattick, MA). Briefly, the local level of background noise was determined and subtracted from dye emission profiles by creating a sub-image (20×20 pixel), followed by calculating the mean photon count rate of the 20 darkest pixels within this area, a method to locally determine background noise adapted from the commonly used aperture photometry approach [3], [59]. Fluorescence time traces were further corrected for leakage of Cy3 emission into the Cy5 channel (∼7%, determined experimentally). Emission time traces were manually selected for anticorrelation and stable acceptor emission to calculate time-dependent apparent FRET efficiencies *FRET(t)* as.
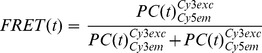
(1)where 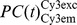 denotes the Cy3 photon count rate upon Cy3 excitation, and 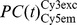 stands for Cy5 emission upon Cy3 excitation.

### Characterization of the Thermodynamic Equilibrium

To characterize the thermodynamic equilibrium, *n* individual FRET time traces *FRET(t)_i_* were binned to 1D histograms *θ_i_(FRET)* using a binning interval of 0.01 FRET units, yielding *m* individual FRET bins. Subsequently, a normalized cumulated FRET histogram was created for all smFRET data recorded under identical imaging conditions:
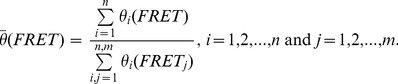
(2)


While individual time traces may be inconclusive in some cases depending on the observation time, the conformational interconversion kinetics, the SNR and the complexity of the system, distinct FRET distributions will develop in the cumulated FRET histogram if discrete conformational species are present and resolvable [3]. The relative occurrence of these states was then quantified by thresholding or multiple Gaussian fitting (Eqs. (13) and (S5)). In threshold-based analysis, the occurrence is quantified by the integral over the area of the cumulated FRET histogram that is assigned to one conformation. For this purpose, the integration limits are defined as −∞, *th_1_*, …, *th_n_*, +∞, where *th* refers to a threshold. Without a loss of generality, we defined the threshold value to distinguish two FRET distributions A and B as (*FRET*_A_+*FRET*_B_)/2, which corresponds to the midpoint between their centers *FRET*_A_ and *FRET*_B_.

Characterization of the thermodynamic equilibrium was also performed using dwell times. The underlying principle is that the time the molecules spend in different discrete states can be directly used to infer the position of the conformational equilibrium. For d3'EBS1*/IBS1*, the docked fraction was used to calculate the association constants *K*_a_ as described in the Supplementary Information S1 (Eqs. (S1) and (S2)). The approaches used to determine dwell times and subsequent processing steps are outlined in the next section.

### Characterization of Kinetics

Dwell times were determined from individual time traces *FRET(t)_i_* via thresholding at (*FRET*_A_+*FRET*_B_)/2 or using the freely available software vbFRET [25]. In short, vbFRET employs a maximum evidence (ME) approach for model selection (the number of FRET states *L*), followed by inferring the model parameters (FRET values and transitions) by a combination of variational Bayesian expectation maximization and hidden Markov modeling (HMM) [60]. As their duration was unknown, the first and the last dwell time of each time trace were consistently discarded. Additionally, a weighted k-means algorithm was applied to transition density plots (TDP) created from the vbFRET data to cluster the coordinates (*FRET*_before transition_; *FRET*_after transition_) into *k* subgroups and assigned each transition to one of the *k* centers (< *FRET*_before transition_ >*_k_*,<*FRET*_after transition_ >*_k_*). The principle of k means clustering is illustrated in Figure S1 and is a well-precedented approach to cluster data that has been applied to heterogeneous HMM data previously [61], [62].

For single-exponential state-to-state transitions occurring in a stochastic manner with rate constants that do not vary over time, *k* subgroups in the TDP correspond to *L* FRET states with *k* = *L* ^2^ − *L*. The corresponding dwell times are in this case exponentially distributed [26]. Consequently, dwell times were binned to histograms that then were used to calculate the normalized cumulative probability distributions 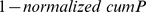, which were in turn fitted to exponential decay functions to extract the corresponding rate constants [11]–[13]. Here, single- and stretched exponential decays were used to approximate simulated and experimental data [14], [34], [63]:
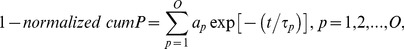
(3)

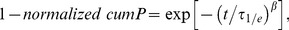
(4)where *O* denotes the number of exponential decays (single-exponential: *O* = 1), *a_p_* is the amplitude, and *τ_p_* the average dwell time in the conformational state (decay constant). The decay time *τ*_1/e_ refers to the time required for 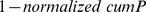 to drop to 1/e of its initial value and the stretching exponent 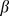 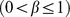 is a means to quantify the width of the rates distribution [64]. Both *τ_p_* and *τ*_1/e_ were used to determine the rate constants associated with conformational changes as described in the Supplementary Information S1 (Eqs. (S3) and (S4)).

### Bootstrapping in Thermodynamic and Kinetic Analysis of smFRET Data

Following the conventions in the field, the variability of the data vector is assumed to be due to limited observation time, experimental noise, instrumental aberrations (heterogeneous broadening, *vide supra*), and irresolvable molecular motion [8], [25]. Bootstrapping allows to characterize the data space of an ensemble of smFRET time traces, and thus, to quantify cross-sample variability and allowing for its application in statistical hypothesis testing.

Bootstrap samples were built for a multi-sample problem given by a random sample of *n* smFRET time traces, each of which is composed of a discrete number of time bins *B* {*FRET(t)*_1_, *FRET(t)*_2_, …, *FRET(t)_n_*}, observed from a completely unspecified probability distribution 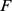 according to Efron [44]. The ensemble of time trajectories were used to create the corresponding single molecule FRET histograms {*θ*_1_ *(FRET)*, *θ*_2_ *(FRET)*, …, *θ_n_(FRET)*}. Resampling was then performed with replacement, where each single-molecule time trace 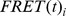 has a probability of.
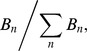
to be selected. Here, *B_n_* denotes number of time bins of the *n*th individual FRET time trace, and the whole expression can be regarded as a weighting factor that accounts for differences in length of individual time traces. Subsequently, bootstrap samples (boba) were created from previously selected time traces 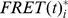 and FRET histograms 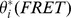:



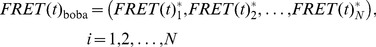
(5)

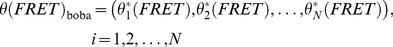
(6)where *N* was set to *n* to prevent pseudoreplication [45]. It should be emphasized, that in the case of an equal length of the time traces (constant observation time, no photobleaching etc.) the probability simplifies to 1/*n*, *i.e.* each time trace and its corresponding histogram has the same probability of being selected (molecular weighting). Normalized cumulated FRET histograms of the bootstrap-based ensemble were calculated as:



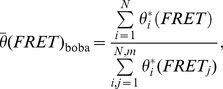
(7)using a Monte Carlo method to approximate the bootstrap distribution with a random sample of the size *N*, the creation of bootstrap samples was repeated *M* times, yielding an independent random ensemble of bootstrap time traces 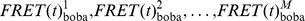, as well as the corresponding histograms 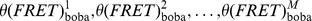 and normalized cumulated FRET histograms 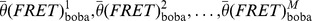. The bootstrap mean 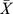
_boba_ and the corresponding standard deviation 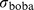 were estimated according to [50]:
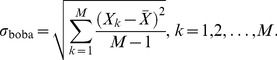
(8)


Here, *X* denotes the random parameter whose variability is to be estimated, for example the relative occurrence of a certain FRET population *A_l_* given by a certain state *l* in the thermodynamic analysis.

The bootstrap distribution of 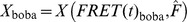, depends on both the random sample 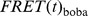 and the sample probability distribution 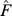. *X*_boba_ is expected to approximate the real underlying distribution 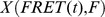 well, including its mean and standard deviation. In this study, we chose *M* = 100, following the conventions from other fields [45], because a time-consuming increase of *M* would yield only moderate improvements (Figure S2) [44]. It is important to emphasize that the noise-induced fluctuation around discrete values in smFRET time traces is entirely time-independent (stochastic). This is not always the case for time series, which would then require more sophisticated mathematical treatments (Figure S3) [44], [65].

### Bootstrapping and Regression (Method 1)

To estimate the bootstrap mean 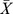
_boba_ and the standard deviation *σ*_boba_ of the parameter *X*, we defined a reasonably general non-linear regression model:
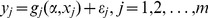
(9)where *g* denotes a model function of the unknown parameter vector *α* approximating the data vector *y* (outcome variable) depending on *x* (input variable), both of which display the length *m*. The corresponding residuals *ε_j_* follow the unspecific probability distribution *ε_j_* ∼ *F*. We fitted *y* based on a non-linear least square regression to estimate *α* [66]:
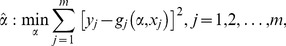
(10)which yields the sampling distribution of 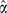. Subsequently, bootstrap samples were generated according to Eqs. (5)–(7) and are henceforth referred to as 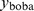 using the terminology of Eq. (9):



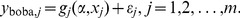
(11)Regression based on a non-linear least square criterion was performed in an analogous manner as in Eq. (10):
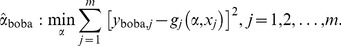
(12)


Applying this procedure on *M* independent bootstrap samples yielded a random sample 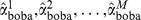 that was used to estimate 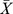
_boba_ and *σ*_boba_. These values were later used for analysis of variance (ANOVA) [66].

The non-linear regression model was then applied to the normalized cumulated 1D FRET histograms 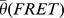 to quantify the variability associated with the determination of thermodynamic parameters. According to the conventions of the field, different conformational states were quantified by multiple Gaussian fitting:



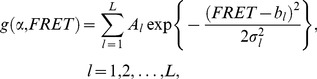
(13)where *L* denotes the number of states that was in our case determined beforehand using a maximum evidence approach (*vide supra*), even though other model selection approaches are conceivable [8], [25]. *A_l_* refers to the respective amplitudes, *b_l_* to the center values, and *σ_l_* to the width of the distribution. The ensemble of model parameters constitute the parameter vector 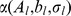. The resulting regression model Eq. (11) for each bootstrap sample is defined as
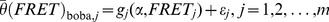
(14)and according to the non-linear least square fitting procedure described in Eq. (12)



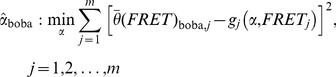
(15)we obtained the representation 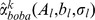 of the sampling distribution 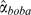.

Second, we applied the bootstrap-based regression on 1 - *normalized cumP* distributions to quantify the variability associated with the analysis of kinetics (*vide supra*, “characterization of kinetics”). The appropriate model function based on Eq. (3) was obtained through the maximum evidence algorithm, which samples the model space as well as the parameter space to find the most evident model and yields the number of components *O* [25]:



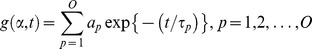
(16).

**Figure 3:**
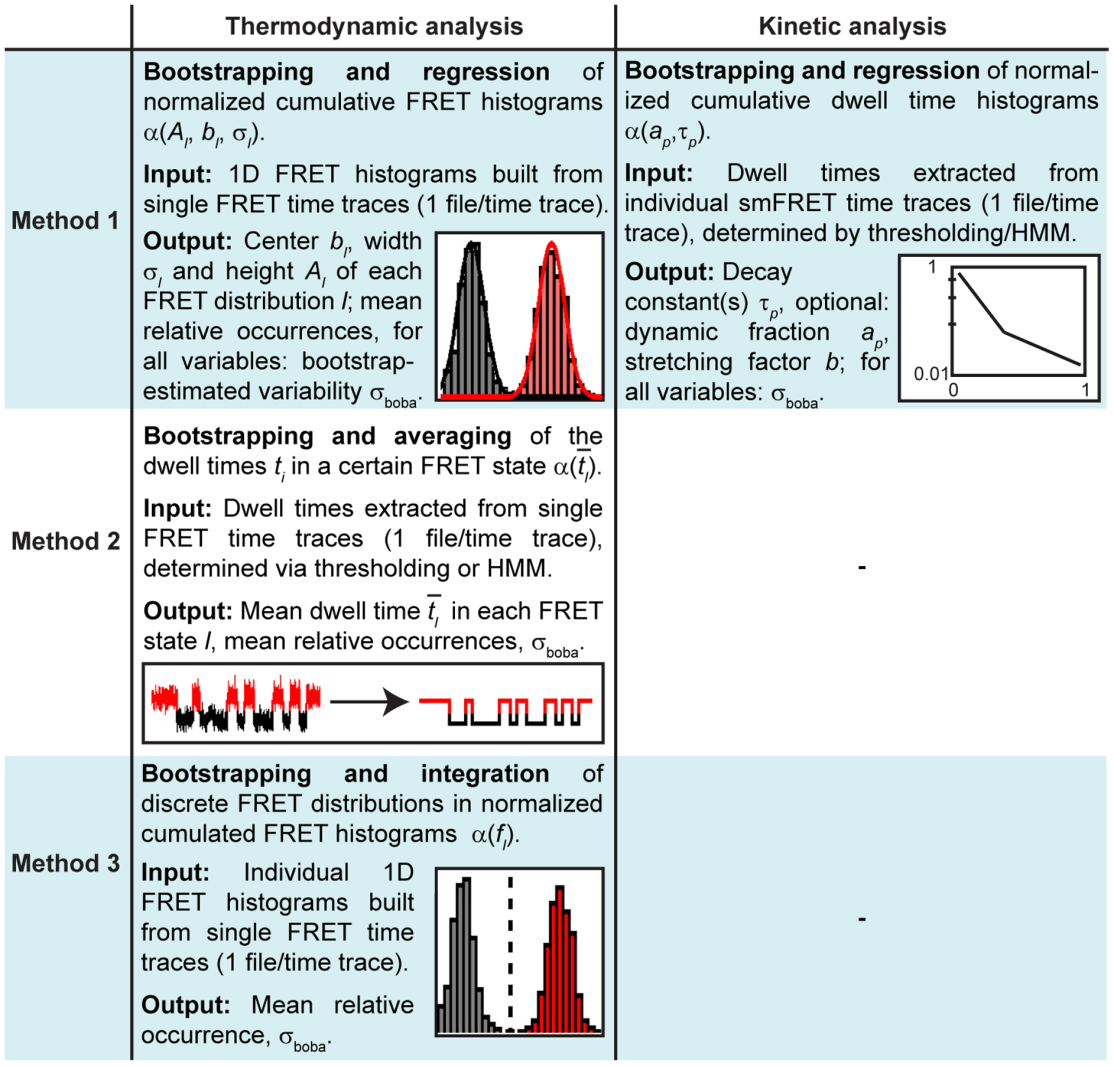
Summary of the different analytical approaches performed in conjunction with bootstrapping to extract thermodynamic or kinetic parameters from time-binned smFRET data in this study. The respective input and output variables are indicated as well. Please refer to the method section for a detailed mathematical description.

Thus, the regression model Eq. (11) for each bootstrap sample was defined as:
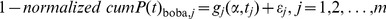
(17)and



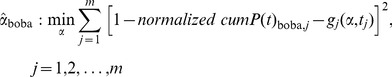
(18)Thus, we obtained the representation 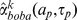 of the sampling distribution 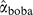. Considerations regarding method 1 are summarized in Figure 3.

### Bootstrapping and Averaging (Method 2)

The bootstrapping formalism described above was also applied in the analysis of the thermodynamic equilibrium using dwell times obtained by threshold- or HMM-based analysis of smFRET time traces. Here, each time trace *FRET(t)_i_* is composed of a number of *m* dwell times *t_i,j,l_* in a discrete state *l*. As a consequence, each bootstrap sample *FRET(t)*_boba_ yields an average dwell time in a certain state.
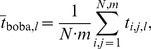
(19)where *i* = 1, 2, …, *N* accounts for the time traces of N different molecules and *j* = 1, 2, …, *m* for the dwell times in the state *l*. Again, applying this procedure on *M* independent bootstrap samples yielded a random sample 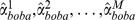 that was used to estimate 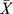
_boba_ and *σ*_boba_ of the thermodynamic parameters. Here, we determined the relative occurrence of each state, as well as the equilibrium constant *K*_eq_ or, in the special case of an intermolecular association of the type A+B ↔ AB, the binding constant *K*_a_ (Eqs. (S1) and (S2)). Considerations regarding method 2 are summarized in Figure 3.

### Bootstrapping and Integration (Method 3)

Finally, we applied bootstrapping on normalized cumulated FRET histograms 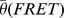 in conjunction with thresholding. Here, each bootstrap sample 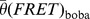 yielded a threshold value (*FRET*_boba, A_+*FRET*_boba, B_)/2 which was used to quantify the relative occurrence of each FRET state as explained before. In an analogous manner, applying this procedure on *M* independent bootstrap samples allowed us to estimate 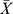
_boba_ and *σ*_boba_ of the relative occurrence of the FRET states. These values were later used for analysis of variance (ANOVA) [66]. Considerations regarding method 3 are summarized in Figure 3.

Resampling and fitting was done with the software package BOBA FRET that is freely available via http://www.aci.uzh.ch/rna/. Please refer to the Supplementary Information (Figures S5 and S6) for an outline of the BOBA FRET user interface and the built-in routines for the analysis of thermodynamic and kinetic data.

## Results and Discussion

### Robustness of the Software and Simulated Data

The robustness of the algorithm and its compatibility with common approaches used for thermodynamic and kinetic analysis was assessed using a simple intramolecular two-state system. Normally distributed noise was added to simulated time traces that were varied in length, separation of the FRET populations, ratio of the rate constants associated with conformational interconversion, and SNR (Figure S4).

#### Thermodynamic characterization of simulated smFRET data

The relative population of FRET states was quantified using four commonly used approaches: Gaussian fitting of normalized cumulated FRET histograms (**method 1**), the ratio of dwell times obtained by either thresholding or HMM (both **method 2**) [25], and fractional integration after thresholding of normalized cumulated FRET histograms (**method 3**), respectively.

**Figure 4:**
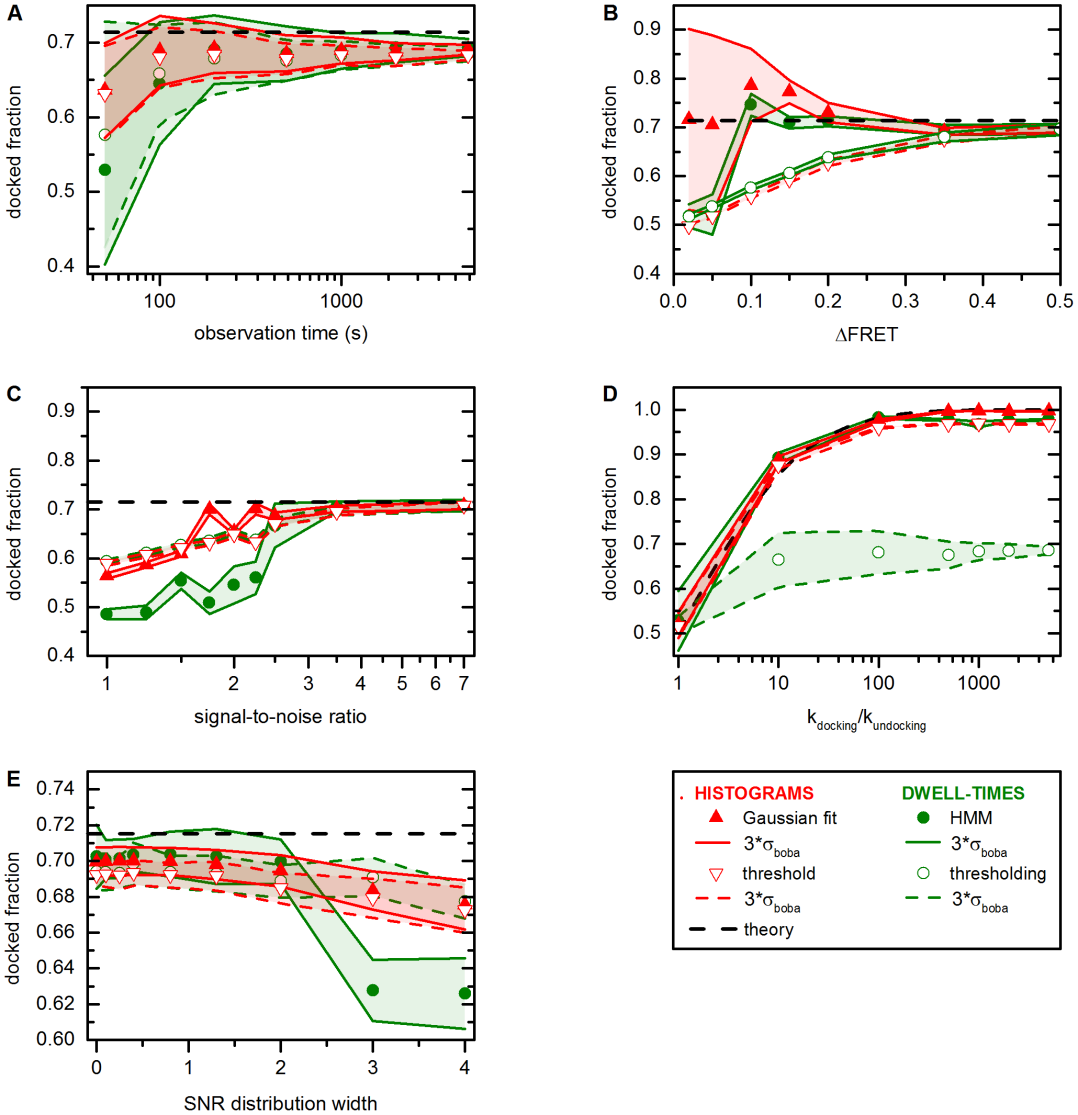
Robustness of different approaches for thermodynamic analysis of smFRET data performed in conjunction with bootstrapping (method 1, 2 and 3, thermodynamics). Simulated data for a two-state system with standard parameters as defined in the methods section. (A) Performance in response to trace length. As the number of data points increases from the left to the right, the mean docked fraction is estimated more precisely, while cross-sample variability decreases. (B–C) Performance in response to FRET spacing and SNR. A systematic downward bias is observed for threshold- and HMM-based approaches as the two FRET distributions show increasing overlap. Gaussian fitting performs well as long as the Rayleigh criterion is fulfilled (*ΔFRET* >0.144). (D) Performance in response to the ratio of rate constants. Threshold-based dwell time analysis easily breaks down, as noise in the docked state is mistaken for FRET transitions. At high *k*_docking_/*k*_undocking_, Gaussian fitting and thresholding of FRET histograms underestimate the docked fraction because of slight overlap between the two FRET distributions. HMM yields the best results. (E) Performance in response to heterogeneously distributed *SNR* values. The results of the threshold-based analysis and Gaussian fitting are mostly unaffected by changes in the SNR distribution width, while HMM breaks down at *σ*(*SNR*) >2. All theoretical values were determined from the input parameters used of the simulations. Error bars (red and green swaths) were estimated by bootstrapping and cover 99.7% of the experimental variability (3*σ*_boba_). Please refer to Figure S4 for representative simulated time traces and the text for further details.

Figure 4A demonstrates that the estimation of the docked fraction becomes more accurate at longer observation times. At the same time, the bootstrap-estimated error scales inversely to the length of time traces. This is expected, as longer time traces yield more data points. Dwell-time-based methods perform poorly at short observation times, because the data before the first transition and preceding the last one are discarded. Importantly, the bootstrapped variability faithfully covers the theoretical values.

Figure 4B shows the influence of FRET spacing (*ΔFRET*) on 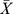
_boba_ and *σ*_boba_. In general, threshold-based approaches lead to a systematic downward shift of the estimated mean and estimations of cross-sample variability that do not cover the predicted values at low *ΔFRET* values. Similarly, HMM does not reliably distinguish the docked from the undocked state at *ΔFRET* <0.1. In turn, Gaussian fitting provides good estimations of the docked fraction, albeit *σ*_boba_ is considerably more pronounced than for other methods at low *ΔFRET* values. The same trend is observed with decreasing SNR (Figure 4C). As *ΔFRET* and *SNR* diminish, the two FRET distributions get closer, becoming indistinguishable in extreme cases (Figure S4), explaining the bad performance of thresholding and why this approach should not be employed under these circumstances (Figure 1). HMM sets somewhat lower standards to the separation of the FRET distributions, though, it erroneously suggests equal population of both FRET states once it breaks down. Finally, even though the results of the Gaussian fits are biased by large error bars when the Rayleigh criterion is not fulfilled, the means are in excellent agreement with the theoretical values.

Figure 4D illustrates how the mean docked fraction and the cross-sample variability depend on the ratio of rate constants. Here, only the docking constant *k*_docking_ is increased, while *k*_undocking_ is kept constant at 0.005 s^–1^, leading to a decreased average number of FRET transitions per time trace. As Gaussian fitting does not rely on faithful determination of dwell times, it provides an excellent estimation of the mean docked fraction and low cross-sample variability. In turn, threshold-based histogram analysis, HMM, and in particular threshold-based dwell time analysis underestimate the docked fraction when the thermodynamic equilibrium favors one conformation. Careful analysis of the FRET distributions from simulated smFRET time traces revealed that at SNR 3.5, the noise exceeds the threshold at times, explaining issues associated with thresholding. This is particularly problematic in the case of threshold-based dwell time analysis, as the ratio of false and true transitions then becomes highly unfavorable. In turn, when a conformational state is very scarcely populated, the mean dwell time becomes shorter than the time resolution and HMM fails to identify two FRET populations.

Figure 4E depicts the variation of 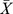
_boba_ and *σ*_boba_ depending on the width of a SNR distribution, *i.e.* assuming intermolecular heterogeneity with regard to SNR within one dataset. For this purpose, SNR was assumed to be normally distributed around 3.5 and the width of the Gaussian distribution was varied between 0 (no heterogeneity) and 4 (strong heterogeneity). Analysis of FRET histograms and threshold-based dwell time analysis systematically under-estimate the mean bound fraction by 3–5%, which is due to the overlap between the two FRET states (*vide supra*). In turn, HMM-based dwell time analysis yields mean values and cross-sample variabilities that closely approach/cover the theoretical value in the case of narrow SNR distributions. However, as more low SNR time traces are included in the analysis, HMM perform increasingly poorly (*vide supra*). Interestingly, regardless of the method chosen for analysis, the estimation of the cross-sample variability remains mostly unaffected by a change in the width of the SNR distribution.

#### Kinetic characterization of simulated smFRET data

When smFRET time traces display “discrete hops”, *i.e*. consist of piecewise constant signal, rate constants can be extracted from dwell time histograms (Figure 1) [21], [26]. Here, bootstrapping is applied to dwell times obtained by thresholding and HMM, followed by fitting the experimental data to a single-exponential decay model (both method 1, Eq. (3), *O* = 1) [25].

**Figure 5:**
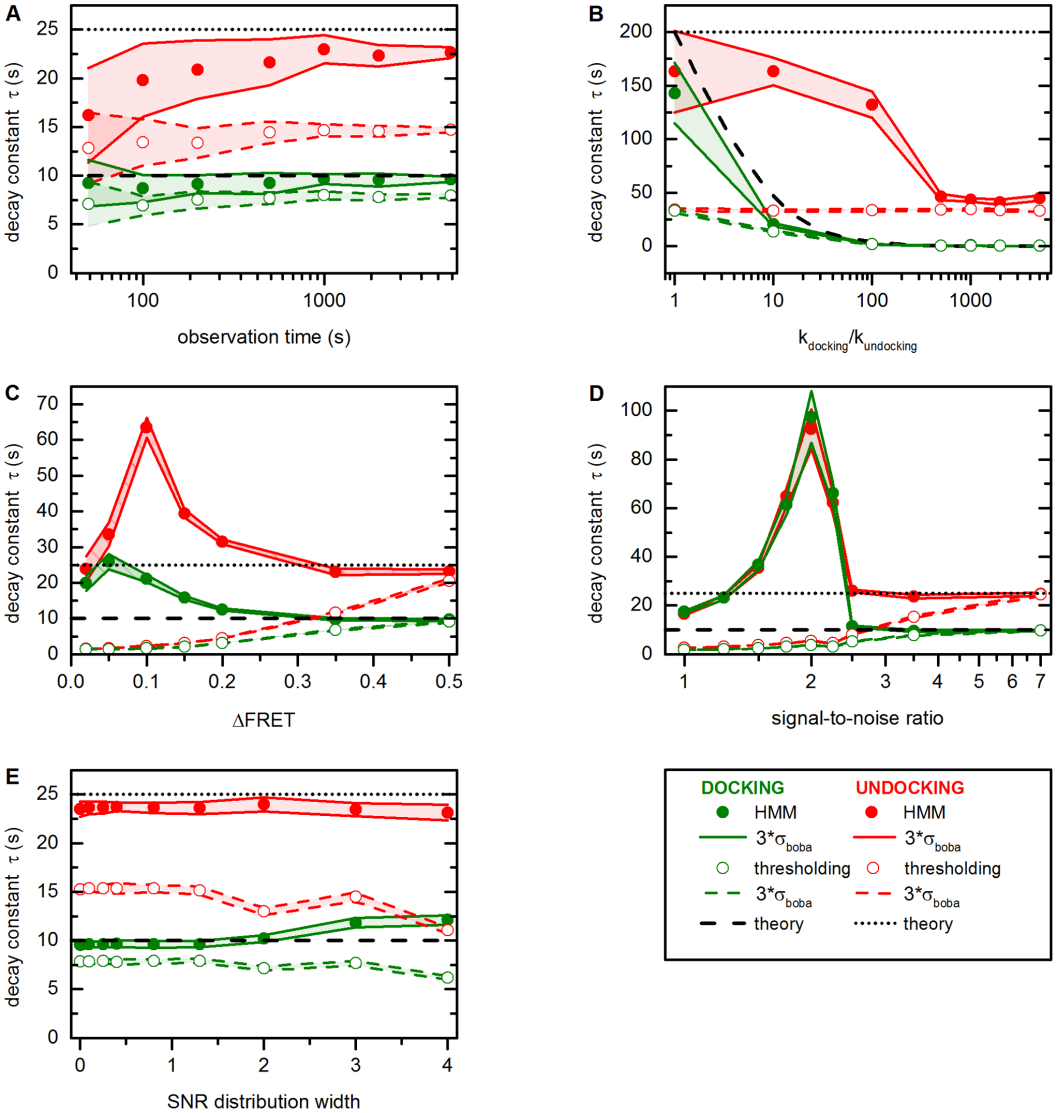
Robustness of thresholding and HMM approaches to analyze smFRET data performed in conjunction with bootstrapping (method 1, kinetics). Simulated data for a two-state system as defined in the methods section. (A) Performance in response to trace length. Cross-sample variability decreases at long observation times, since the number of dwell times increases. (B) Performance in response to the ratio of rate constants. Two problems bias threshold- and HMM-based analysis: (i) false FRET transitions stemming from noise and (ii) irresolvable FRET transitions. (C–D) Performance in response to FRET spacing and SNR. A systematic downward bias is observed for threshold-based analysis as the two FRET distributions show increasing overlap. The result of the HMM-based analysis depends on the ratio of false and true dwell times. (E) Performance in response to heterogeneously distributed SNRs. The results of the threshold-based analysis and Gaussian fitting are mostly unaffected by changes in the SNR distribution width. All theoretical values were determined from the input parameters used of the simulations. Error bars (red and green swaths) correspond to the standard deviation estimated by bootstrapping (3*σ*_boba_). Please refer to Figure S4 for representative simulated time traces and the text for further details.

Figure 5A demonstrates that cross-sample variability strongly decreases when the observation time is increased from 50 s to 5000 s. Again, this is not surprising, as the average number of dwell times per time trace is expected to be proportional to the observation time, which leads to a more homogeneous behavior between individual time traces. However, thresholding systematically underestimates the mean decay constant associated with docking and undocking, an issue that noise is frequently mistaken as a FRET transition at *SNR* = 3.5 (*vide supra*). This problem persists in HMM-based analysis, though, the algorithm proves more robust than thresholding.

The dependence of 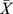
_boba_ and *σ*_boba_ on the number of dwell times is also depicted in Figure 5B, which shows the influence of the ratio of rate constants on the outcome of the dwell time analysis and the value of the bootstrapped error. Here, two effects lead to an underestimation of the decay constants: (i) Due to the (slight) overlap between the two FRET distributions at *SNR = *3.5 there are short false dwell times stemming from noise (*vide supra*). As the ratio of *k*_docking_ and *k*_undocking_ increases the average number of true dwell times per time trace decreases, while the average number of noise-induced transitions is constant. Consequently, dwell times determined from individual time traces become more homogeneous as well, since false transitions are more and more emphasized. (ii) When the thermodynamic equilibrium strongly favors the docked state, brief excursions to the undocked state become irresolvable (*vide supra*).

Figure 5C and Figure 5D show the influence of overlapping FRET distributions on the outcome of the kinetic analysis. In general, threshold-based analysis is strongly biased as the two FRET distributions display increasing overlap. As *ΔFRET* and *SNR* diminish, the two FRET distributions display increasing overlap and the thresholding algorithm erroneously responds to noise, explaining its bad performance. Furthermore, cross-sample variability decreases, as each time trace (erroneously) yields a very high number of dwell times. The behavior of the HMM algorithm is not as easily explained: When the two FRET distributions display very strong overlap, HMM-based analysis yields approximately equal estimations for both for docking and undocking decay constants. Under such sub-Rayleigh conditions, the FRET distributions are essentially indistinguishable and the HMM algorithm (which assumes Gaussian noise) will approximate each time trace with two equally populated Gaussian distributions. As the two FRET distributions become more distinct, HMM tends to considerably overestimate the decay constants, which can be explained by (i) less artefactual transitions, and (ii) more real transitions. At the same time, however, not all transitions are identified, generally yielding an overestimation of the time a molecule dwells in the docked or undocked state. Further improvement of the data quality finally leads to a correct estimation at *ΔFRET* >0.3 and *SNR* >3.5 and at *ΔFRET* >0.4 and *SNR* >2.5. Importantly, throughout these simulations, the bootstrapped standard deviation is not significantly affected. In conclusion, HMM turns out to be more robust than thresholding in response to increasing overlap, an observation that is in excellent agreement with earlier reports [8].

Figure 5E shows how a variation of the SNR within the same dataset affects the estimation of 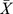
_boba_ and *σ*_boba_. In general the influence of a change in SNR distribution width on both estimators is negligible. Threshold-based analysis consistently under-estimates the values of the decay constants, which stems from the fact that the default signal-to-noise ratio of 3.5 leads to a considerable number of erroneously identified dwell times as described above. In turn, the results of the HMM-based analysis are in good agreements with the theoretical prediction.

Taken together, these simulations illustrate the importance of selecting the correct method to analyze FRET time traces, as the bootstrapping algorithm cannot make up for ill-defined input values. However, when an appropriate approach is chosen, the bootstrapped cross-sample variability generally covers the theoretically predicted mean. Future work is anticipated to develop objective criteria to accept/reject a given model for thermodynamic and kinetic analysis of time-binned smFRET data presented herein.

### Application of the Algorithm to Experimental Data

**Figure 6:**
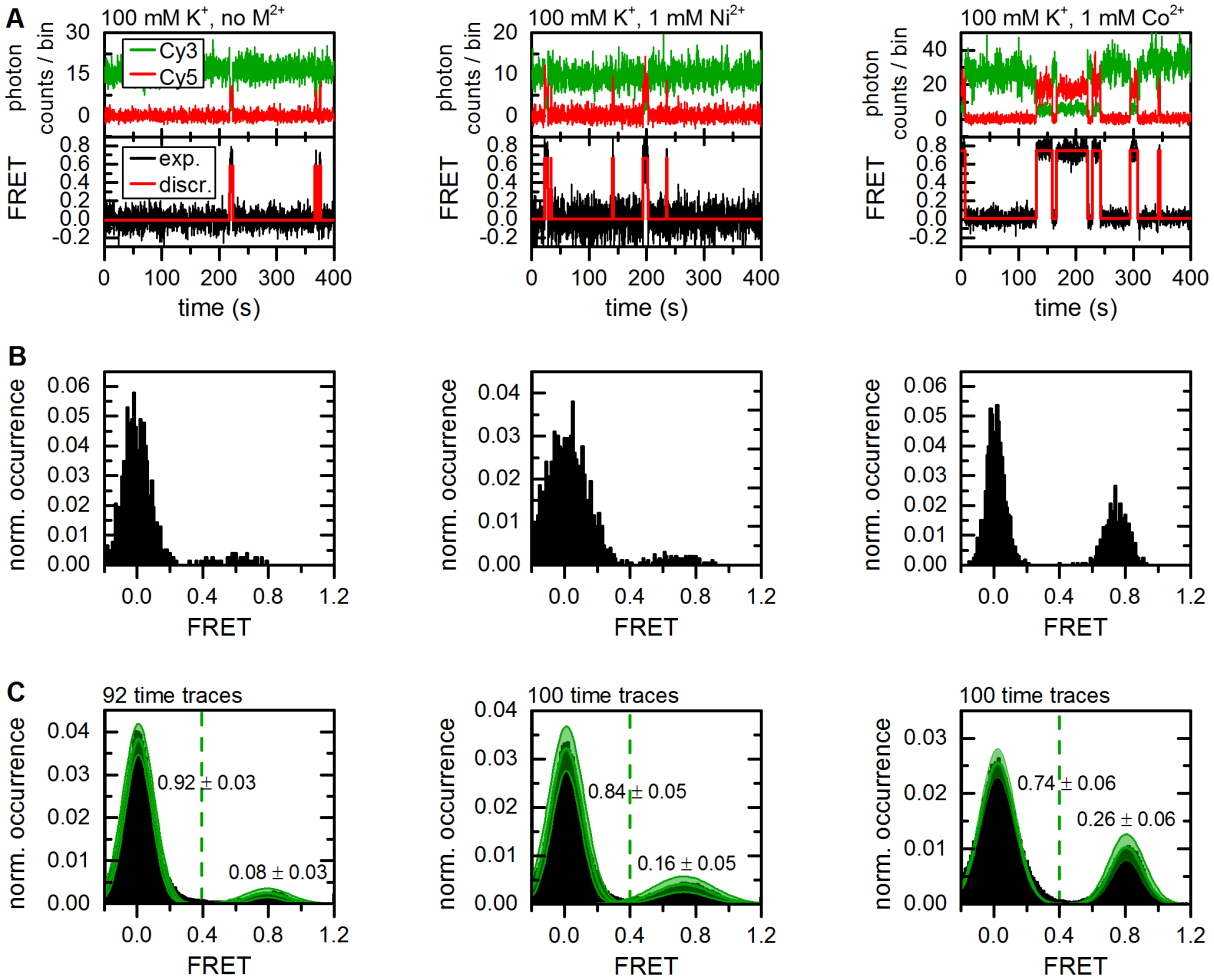
Representative time traces showing d3'EBS1*/IBS* interaction and thermodynamic analysis of FRET histograms (method 1). (A) Fluorophore emission over time reveals abrupt anticorrelated changes in intensity (upper graphs). Calculation of FRET time traces reveals repetitive shuttling between a zero and a high FRET level (lower graphs). Based on the experimental design, these two states were assigned to the undocked and the docked state (Figure 1). The red lines correspond to the discretization by the Hidden Markov Model (vbFRET [25]). (B) FRET histograms built from the smFRET time traces shown in A. (C) Normalized cumulated FRET histograms built from individual time traces. The dashed green line depicts the threshold between the two FRET states used to determine the docked/undocked fractions and the normalized results are indicated. Solid green lines correspond to Gaussian approximation of the experimental data. The error (green swath) is the standard deviation associated with amplitude and width of the Gaussian fit functions as estimated by bootstrapping (3*σ*_boba_).

Time-binned smFRET data have been recorded and analyzed from numerous biological systems varying in size and complexity. Here, we studied an important element derived from the 5' splice site recognition complex of the yeast group II intron *Sc.ai5γ*, the sequence pair d3'EBS1*/IBS1* [51], [53]. As depicted in Figure 2, Cy3- d3'EBS1* strands were tethered to the surface of a quartz slide passivated with biotinylated BSA, while Cy5-IBS1* molecules were free in solution. Hence, docking/undocking dynamics could be followed via FRET over several minutes and in the presence of different divalent metal ions, as splice site formation has previously been proposed to depend on the action of divalent metal ions [67]. FRET-typical anticorrelated changes in Cy3 and Cy5 emission intensity were observed in all cases, followed by calculating the *FRET* over time (Eq. (1)), which varied between zero (undocked) and a high *FRET* value (docked) for all dynamic molecules observed (Figure 6A). The fraction of statically undocked molecules, *i.e.* molecules that displays only donor emission during the time of observation, was 60% in the absence of M^2+^ and 20% in the presence of Ni^2+^ or Co^2+^. This fraction of molecules either displays a low association constant *K*_A_ that cannot be correctly resolved during the observation time and/or they correspond to a photophysical artifact, for example a docked IBS1* molecule with a non-emissive acceptor [42]. In fact, 15–55% of the total population is usually “donor only” in smFRET studies using the FRET pair Cy3 and Cy5, which has been attributed to Cy5 pre-bleaching [68]. As a consequence, these molecules were excluded from further analysis.

#### Divalent metal ions have a significant effect on the thermodynamic equilibrium

**Figure 7:**
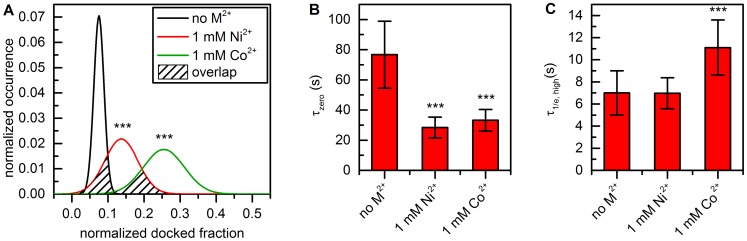
Statistical hypothesis testing using thermodynamic and kinetic smFRET data. (**A**) Analysis of variance (ANOVA) of docked fractions determined by thresholding of normalized cumulated FRET histograms (Figure 6C) reveals that Ni^2+^ and Co^2+^ shift the conformational equilibrium significantly towards the docked state (*** P<0.001). The outcome of an ANOVA depends on the separation of the means (center values of the Gaussians) and how far the values are spread out (variance, *σ*_boba_^2^, width of the Gaussians) and is given in form of a P-value, *i.e*. the probability that the null hypothesis is true (here: divalent metal ions do not significantly affect the equilibrium). The stronger the overlap between different groups, the greater the P-value. (**B**) Decay constants of the zero FRET state decrease in response to addition of Ni^2+^ or Co^2+^, leading to faster association (P<0.001). Data obtained by HMM analysis and single-exponential fitting (Figure 8). (**C**) Decay constants of the high FRET state significantly increase in the presence of Co^2+^ (P<0.001), which promotes stable association of d3'EBS1* and IBS1*. Data obtained by HMM analysis and stretched exponential fitting (Figure 8). Error bars correspond to the bootstrapped standard deviation (3*σ*_boba_).

Bootstrapping was performed in conjunction with Gaussian fitting (**method 1**) and thresholding (**method 3**) of normalized cumulated FRET histograms (Figure 6B, C). The thermodynamic equilibrium was also characterized using dwell times obtained by HMM (**method 2**) [25]. Threshold-based analysis reveals weak inter-oligonucleotide interaction in the absence of divalent metal ions (docked fraction: 7.8±2.6%, errors correspond to 3*σ*_boba_ unless specified differently, Figure 6C and Table 1). Addition of 1 mM Ni^2+^ shifts the equilibrium slightly (docked fraction: 16.3±3.3%), while an average of 25.5±6.0% of all d3'EBS1* molecules are docked to IBS1* at 1 mM Co^2+^. One-way analysis of variance (ANOVA) using bootstrapped values was performed to test the hypothesis that divalent metal ions affect or do not affect (null hypothesis) the thermodynamic equilibrium [66]. As illustrated in Figure 7A, an ANOVA makes the assumption that experimental values are normally distributed around the sample mean and its outcome (P-value) depends on the overlap integral between different distributions, which in turn depends on the separation of group means and the widths of the sample distributions. P-values constitute a strength of evidence against the null hypothesis and are typically compared to arbitrary values (0.05, 0.01 and 0.001) according to the conventions of the field [66]. The presence of divalent metal ions not only significantly promotes the interaction of the two oligonucleotides (P<0.001), the effect also differs significantly between Ni^2+^ and Co^2+^ (P<0.001), the latter being much more effective in increasing the docked fraction (Figure 7A). Similar results were obtained by fitting the averaged 1D histograms to two Gaussian distributions (Table 1), though the bootstrap-estimated errors are generally higher (Figure 6C). However, this did not strongly influence the significance of the effect (P<0.001, data not shown). Thermodynamic analysis using dwell times (**method 2**) leads to a systematic shift of the mean docked fraction towards higher values and an increase of *σ*_boba_ (Table 1). Nevertheless, the results of all methods are generally in good agreement (Table 1).

**Table 1:** Thermodynamic analysis of the d3'EBS1*/IBS1* equilibrium by different methods.

	Imaging condition	Fraction of docked d3'EBS1* (%)	*K*_a_ (L µmol^−1^)
**Gaussian fitting (method 1)**	no M(II)(NO_3_)_2_	8.2±3.0	3.6±1.3
	1 mM Ni(NO_3_)_2_	18.5±6.1	9.1±3.7
	1 mM Co(NO_3_)_2_	28.6±5.9	16.1±4.6
**Dwell time analysis (HMM, method 2)**	no M(II)(NO_3_)_2_	15.6±6.4	7.4±3.6
	1 mM Ni(NO_3_)_2_	24.6±6.3	13.1±4.5
	1 mM Co(NO_3_)_2_	33.3±8.4	20.1±7.5
**Histogram thresholding (method 3)**	no M(II)(NO_3_)_2_	7.8±2.6	3.4±1.2
	1 mM Ni(NO_3_)_2_	16.3±5.4	7.8±3.1
	1 mM Co(NO_3_)_2_	25.5±6.0	13.7±4.5

The experimental error was estimated by bootstrapping and accounts for 99.7% of the variability observed (3*σ*_boba_, “68–95–99.7 rule” [66]). Association constants *K*_a_ were calculated from normalized cumulated FRET histograms or dwell times under the assumption that [IBS] = [IBS1*]_tot_ as described in the Supplementary Information S1 (Eqs. S1 and S2).

Taken together, the results of histogram and dwell time analysis are in good agreement and demonstrate the significant role of low concentrations of divalent metal ions in shifting the thermodynamic equilibrium of d3'EBS1* and IBS1*. However, a systematic upward shift of the estimation of the docked fraction is observed that is most pronounced in the absence of divalent metal ions. These findings demonstrate that the dwell time approach has to be employed with care, especially when the biomolecule is poorly dynamic (60% of statically undocked molecules in the absence of M^2+^, *vide supra*) and/or the number of dwell times is rather low, two problems that are often linked. Indeed, the average number of dwell times per time trace was less than 4, which contrast the average value of the simulations carried out using standard parameters (114, *vide supra*). As the first and the last dwell time were not considered (*vide supra*), (i) much information was lost leading to an increase in the bootstrapped error and (ii) the occurrence of the more populated undocked state is underestimated translating into higher values of the docked fraction. Bias of dwell-time based approaches in the case of low numbers of dwell times can also be seen in the simulations (Figure 5A).

#### Divalent metal ions significantly alter d3'EBS1*/IBS1* interaction kinetics

**Figure 8:**
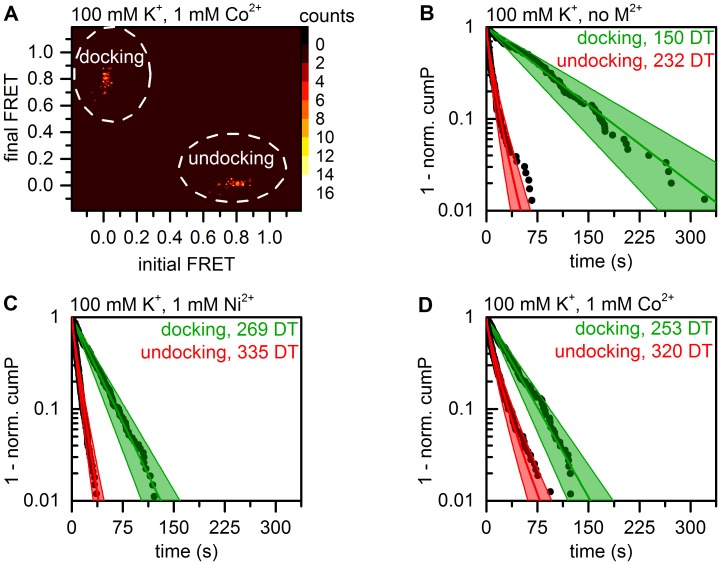
Kinetic analysis of smFRET data (method 1). Docking is defined as the state transition from the undocked to the docked state, the undocking process is defined as the inverse reaction. (**A**) Transition density plots of HMM data show two clusters corresponding to the docking and the undocking reaction, respectively. According to the maximum evidence approach employed in vbFRET [25], a two-state system is therefore most likely to produce the experimental data, which is in agreement with the experimental design. Raw data were grouped via the weighted k-means clustering algorithm. Color code: occurrence in counts. (**B**–**D**) Dwell time histograms created from the normalized cumulative occurrence of dwell times in the docked and the undocked state as determined by HMM. The green lines correspond to a single-exponential fit to the experimental data, while the red lines represent a stretched exponential decay. Errors are indicated as a swath and correspond to 3*σ*_boba_ associated with the decay constants.

d3'EBS1*/IBS1* dissociation has previously been shown to display considerable kinetic heterogeneity in the presence of divalent metal ions [33], [69], [70]. As a consequence, a stretched exponential decay (Eq. (4)) was fitted to dwell times in the high FRET state, while a single-exponential decay (Eq. (3), *O* = 1) was used to approximate the association kinetics. Dwell times were determined from individual time traces using thresholding and HMM, followed by clustering of transition density plots using a weighted k-means algorithm (Figures 8A and S1) [5], [25]. Then, cumulative probability plots *cumP* were created from dwell times, followed by fitting 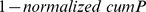 plots to exponential decay functions (Figure 8B–D). Dwell times were resampled via bootstrapping (**method 1**) to estimate the variability of the decay constants and the stretching parameter *β*.

HMM-based data on interstrand association is well described by single-exponential fit in the absence of divalent metal ions (Eq. (4), *β* = 0.99 data not shown) and the process was found to occur very slowly (*τ*_docking_ = 76.7±22.2 s). Both the presence of Ni^2+^ and Co^2+^ accelerates this reaction, albeit to different extents (*τ*_docking_ = 28.4±6.1 s and 31.6±7.3 s). These metal-ion-specific effects are highly significant as shown by one-way ANOVA (P<0.001, Figure 7B). Importantly, the presence of divalent metal ions also induces slight broadening of the distribution of observed association rates (*β*(Ni^2+^) = 0.95, *β*(Co^2+^) = 0.95, data not shown), though the experimental data could nonetheless be satisfactorily approximated with the single-exponential fit (adjusted R^2^>0.98 in all cases). d3'EBS1*/IBS1* dissociation is fast in the absence of divalent cations (*τ*_undocking, 1/e_ = 7.0±1.9 s). Co^2+^ significantly slows down the dissociation rate (*τ*_undocking, 1/e_ = 10.0±2.7 s, P<0.001), while the presence of Ni^2+^ does not induce any variation in the decay constant (*τ*_undocking, 1/e_ = 7.0±1.4 s, Figure 7C). In agreement with previous observations, the distributions of decay constants are severely broadened (*β* <0.9 in all cases), underscoring the kinetic heterogeneity of the undocking process. The results of the threshold-based analysis are generally in excellent agreement with the values obtained from fitting HMM-derived dwell times. However, the decay constant associated with docking in the absence of divalent metal ions display a difference of 70%. All results are summarized in Table 2.

**Table 2:** Kinetic analysis of d3'EBS1*/IBS1* association and dissociation using different methods to extract dwell times.

	Imaging condition	*τ*_docking_ a(s)	k_docking_(s^−1^ µM^−1^)	*τ*_1/e,undocking_^b^ (s)	*β*	k_undocking_ (s^−1^)
**Thresholding (method 1)**	no M(II)(NO_3_)_2_	44.9±26.8	0.93±0.60	7.6±3.0	0.79±0.08	0.156±0.090
	1 mM Ni(NO_3_)_2_	31.7±7.3	1.27±0.29	6.2±1.3	0.79±0.04	0.175±0.045
	1 mM Co(NO_3_)_2_	31.7±7.9	1.27±0.31	10.9±2.6	0.75±0.04	0.110±0.033
**Hidden Markov modeling (method 1)**	no M(II)(NO_3_)_2_	76.7±22.2	0.53±0.15	7.0±2.0	0.77±0.05	0.168±0.057
	1 mM Ni(NO_3_)_2_	28.4±6.1	1.41±0.31	7.0±1.4	0.88±0.05	0.154±0.042
	1 mM Co(NO_3_)_2_	33.2±7.2	1.21±0.26	11.1±2.5	0.78±0.04	0.105±0.027

The experimental error was estimated by bootstrapping and accounts for 99.7% of the variability observed (1*σ*_boba_ for *β*, 3*σ*_boba_ in all other cases). Rate constants were calculated as described in the Supplementary Information S1 (Eqs. (S3) and (S4)).

^a^ Single-exponential fit, ^b^Stretched exponential fit.

These findings suggest that the presence of divalent metal ions broadens the distribution of rate constants associated with d3'EBS1*/IBS1* interaction. Based on the NMR structure and metal ion titration studies of the d3'EBS1* hairpin in the absence and presence of IBS1*, this effect has been assigned to heterogeneous occupation of metal ion binding sites along the RNA [70]. Such kinetic heterogeneity is beyond the scope of conventional kinetics and has frequently been observed in single-molecule experiments [33], [43]. In the context of this paper, kinetic heterogeneity contrasts the basic assumption made in first-order HMM, *i.e.* that state-to-state transitions are governed by single-exponential kinetics. The ability to assign one FRET level to multiple Markov transition rates is therefore important, an important feature that is implemented in some HMM software packages (vbFRET, CSSR) but not others (HaMMy) [8], [25], [27].

Fitting exponential decay models to bootstrapped dwell time histograms also permitted to show that changes in both association and dissociation kinetics are highly significant. Taken together, Ni^2+^ shifts the thermodynamic equilibrium chiefly by promoting the association rate, while Co^2+^ plays a two-fold role as an accelerator of docking and as an inhibitor of dissociation, probably by mediating specific contacts between the two RNA fragments. This difference is surprising, as both metal ions share very similar ionic radii (Ni^2+^: 0.83 Å, Co^2+^: 0.79 Å) and have the same preferred coordination geometry (octahedral, 6 ligands) [71]. Fits of threshold- and HMM-based dwell time data were generally in good agreement, except for docking in the absence of divalent cations. Careful analysis of HMM data revealed that brief excursions to the docked state were not always identified as such, especially when very few and short binding event occurred in the time trace (data not shown). Instead, the zero FRET distribution was erroneously identified as two distinct states. This observation contradicts the simulations and is most likely due to the fact that noise in experimental time traces does not always follow a stochastic Gaussian model (Figure S3). These findings suggests that HMM approaches are not always the best choice for analyzing smFRET data, in particular when one conformation largely dominates the structural equilibrium and the occurrence of other structures may be erroneously deemed statistically insignificant by the HMM algorithm and non-Gaussian noise is fitted instead. As binding events became more frequent and/or long-lasting, HMM and thresholding were found to be in very good agreement.

## Summary

Single-molecule FRET has led to valuable work on mechanistic and structural aspects of numerous biological processes and has blossomed in recent years. However, the observation time of single fluorophore emission is rather limited, as dyes typically photobleach upon emission of 10^6–^10^7^ photons (unpublished data involving Cy3 and Cy5 emission in the presence of an enzymatic oxygen scavenging system and 1 mM Trolox) [72]. Furthermore, the detected signal, intrinsically weak in intensity, is further broadened by various sources of additive noise and technical issues. As a consequence, single molecules typically display considerable cross-sample variability and can then not be treated as biological replicates in thermodynamic and kinetic analyses, *i.e*. rate and association constants cannot be inferred from individual smFRET time traces. In such cases, smFRET relies on the principle of ergodicity, according to which the properties of ensembles involving billions of molecules be described by combining a number of single molecules that is lower by several orders of magnitude [73]. Analogously, bootstrapping computes the distribution of the whole population, including measures of variance, from a sample distribution of the size *n* [51].

Herschlag and co-workers have recently recognized the need for statistical rigor in smFRET experiments and implemented an HMM algorithm that assigned confidence intervals to rate constants inferred from individual time traces [26], [74]. Thus, one can investigate whether kinetically distinct subspecies exist within the sample, a long-standing topic of debate in the field of single-molecule spectroscopy [33], [43]. However, this approach sets very high standards to the data, as the confidence interval scales inversely to the number of transitions in the FRET time trace, and simulated time traces in the original article were composed of up to 5'000 dwell times [26]. Given the technical constraints outlined above, these values may be difficult to reach experimentally. Here, we have combined bootstrapping with different approaches commonly used in thermodynamic and kinetic analysis of smFRET data in order to estimate the variability associated with the mean values. By performing statistical hypothesis testing using generalized analysis of variance (ANOVA), we could show that divalent metal ions have a statistically significant effect on both thermodynamics and kinetics of d3'EBS1*/IBS1* interaction, a pair of RNA sequences involved in group II intron splice site recognition. Importantly, the fact that time traces were on average composed of only 4–6 dwell times was not problematic, since the overall data was treated as an ensemble according to the principle of ergodicity. We therefore believe that this approach is widely applicable and it is expected to make biological interpretations in smFRET experiments more robust when it is combined with statistical testing. Finally, it should be mentioned that the method described herein is not limited to time-binned smFRET data. We anticipate its implementation to analyze time traces stemming from single photon detection. A further potential application is the characterization of conformation and orientation dependent fluorophore photophysics (blinking, spectral and spatial diffusion) [75]–[77].

BOBA FRET was developed under Matlab version 8.20.701, license 49040 (Mathworks, Nattick, MA) and is available at http://www.aci.uzh.ch/rna/. Some of the data presented herein are provided for download as well.

## Supporting Information

Figure S1 **k-means clustering to assign dwell times to consistent FRET values for further processing steps.** (**A**) Transition density plot (TDP) built from a set of HMM-discretized FRET time traces. The data points are iteratively assigned to one of the two centers according to their distance. The center coordinates are then recalculated according to the distances and occurrences (weights) of the clustered data point. The weighted k-mean centers are assumed to be definitive when the set of clustered transition does not change after an additional round of iteration. (**B**) Dwell time analysis of one simulated FRET time trace for a two state system: *ΔFRET = *0.04, *FRET*_A_ = 0.48 (undocked state), *FRET*_B_ = 0.52 (docked state); *SNR* = 6.0 (width *σ* = 0.143); observation time = 4000 s (magnified to highlight transitions); *k*_docking_ = 0.04 s^–1^ (intramolecular reaction) *k*_undocking_ = 0.1 s^–1^. Each of the two FRET states detected in the trace are assigned to the center of one of the two clusters and the corresponding dwell times are subsequently used for thermodynamic or kinetic analysis.
(TIF)

Figure S2 **Dependence of the bootstrapped estimated cross-sample variability on the number of bootstrap samples.** **(A)** Gaussian fitting was performed in conjunction with bootstrapping to analyze 100 simulated smFRET time traces (*N* = 100, Eq. (8)). The number of bootstrap samples was varied between 5 and 1000 (*M*, Eq. (9)). The histogram corresponds to the normalized cumulated histogram built from all time traces (Eq. (3)), solid lines depict Gaussian fit functions, dashed lines the variability associated with the amplitude and the width (3*σ*_boba_). **(B)** Fraction of docked molecules and cross-sample variability, data from panel (A). Error bars correspond to 3*σ_boba_. **(C)** Dependence of Δ*σ*_boba_ on the number of bootstrap samples. Data point correspond to the difference in 3*σ_boba_ of adjacent data points and demonstrate that fluctuations become negligible when more than 100 bootstrap samples are used. Parameters of the simulation: *FRET*_A_ = 0.3 (undocked state), *FRET*_B_ = 0.7 (docked state); *SNR* = 3.5; observation time = 100 s; *k*_docking_ = 0.1 s^–1^, *k*_undocking_ = 0.04 s^–1^.
(TIF)

Figure S3 **Statistical nature of noise in smFRET data.** (**A**) Cy3 emission time trace, representative data. Surface-tethered Cy3-tagged d3'EBS1* fluctuates around zero FRET in the absence of IBS1*. (**B**) *FRET*(*t*) versus *FRET*(*t* +100 ms) scatter plot of the data shown in (A) develops as a two-dimensional Gaussian distribution. Time-dependent noise would be expected to accumulate on a diagonal. (**C**) The autocorrelation function of the data in (A) clearly demonstrates that the noise of the time trace shown in (A) is time-independent.
(TIF)

Figure S4 **Representative data of simulated smFRET time traces and normalized histograms, representative data.** Standard parameters of the simulation: *FRET*_A_ = 0.3 (undocked state), *FRET*_B_ = 0.7 (docked state); *SNR* = 3.5; observation time = 4000 s; *k*_docking_ = 0.1 s^–1^, *k*_undocking_ = 0.04 s^–1^. **(A)** The observation time is varied between 50 s and 4000 s (1 frame per second). **(B)** The ratio of rate constants associated with “docking” and “undocking” is changed from 1 to 5000 (*k*_undocking_ = 0.005 s^–1^ = constant; 0.005 s^–1^≤ *k*_docking_ ≤25 s^–1^). **(C)** The spacing of the centers of the FRET distributions is varied from 0.5 to 0.02. **(D)** The signal-to-noise ratio is varied from 7 to 1.
(TIF)

Figure S5 **Boba FRET user interface for thermodynamic analysis.** (**A**) Data import from ASCII files. Both smFRET histogram files (first column: FRET, second column: occurrence (counts); further columns are ignored) and dwell time files are supported (first column: duration, second column: FRET before transition, third column: FRET after transition). (**B**) Optional determination of the optimal number of Gaussians by distribution analysis [79], [80]. (**C**) Setting the parameters for bootstrapping (*N* and *M*, Eqs. (7) and (8) in the main text). (**D**) Setting the starting guesses and boundaries of the Gaussian fits (Eq. (13) in the main text). Alternatively, thresholding can be performed. (**E**) Original normalized data and fitting results. Solid lines correspond to the fit to the original data, dashed lines to the bootstrapped estimated variability (highest and lowest values of the amplitude and the width). (**F**) Goodness of fit to all bootstrapped histograms. All fitting parameters (in the case of Gaussian fitting) and the relative occurrences are automatically exported to text files for further analysis.
(TIF)

Figure S6 **Boba FRET user interface for dwell time analysis.** (**A**) Data import from ASCII files. File format: first column, duration; second column, FRET before transition; third column, FRET after transition. (**B**) Setting the parameters for bootstrapping (*N* and *M*, Eqs. (7) and (8) in the main text). (**C**) Setting the starting values and boundaries of the exponential decay function to be used for fitting. Mono-, bi-, tri-, and tetraexponential decays functions are implemented, as well as stretched exponential decays (Eqs. (3) and (4) in the main text). (**D**) Original normalized data and fitting results. Solid lines correspond to the fit to the original data, dashed lines to the bootstrapped estimated variability (highest and lowest values for the decay constant). All fitting parameters are automatically exported to text files for further analysis.
(TIF)

Information S1 **Supplementary Methods.**
(DOC)

## References

[1] Lakowicz JR (2006) Principles of fluorescence spectroscopy. New York, NY, USA: Springer Science+Business Media, LCC.

[2] Dahan M, Deniz AA, Ha T, Chemla DS, Schultz PG, et al (1999) Ratiometric measurement and identification of single diffusing molecules. Chem Phys 247: 85–106.

[3] Holden SJ, Uphoff S, Hohlbein J, Yadin D, Le Reste L, et al (2010) Defining the limits of single-molecule FRET resolution in TIRF microscopy. Biophys J 99: 3102–3111. 21044609 10.1016/j.bpj.2010.09.005 PMC2965953

[4] König SLB, Liyanage P, Sigel RKO, Rueda D (2013) Helicase-mediated changes in RNA structure at the single-molecule level. RNA Biol 10: 132–147. 10.4161/rna.23507 PMC3590230 23353571

[5] Uphoff S, Gryte K, Evans G, Kapanidis AN (2011) Improved temporal resolution and linked hidden Markov modeling for switchable single-molecule FRET. ChemPhysChem 12: 571–579. 21280168 10.1002/cphc.201000834

[6] Roy R, Hohng S, Ha T (2008) A practical guide to single-molecule FRET. Nat Methods 5: 507–516. 18511918 10.1038/nmeth.1208 PMC3769523

[7] Schuler B, Hoffmann H (2013) Single-molecule spectroscopy of protein folding dynamics - expanding scope and timescales. Curr Opin Struct Biol 23: 36–47. 23312353 10.1016/j.sbi.2012.10.008

[8] McKinney SA, Joo C, Ha T (2006) Analysis of single-molecule FRET trajectories using hidden Markov modeling. Biophys J 91: 1941–1951. 16766620 10.1529/biophysj.106.082487 PMC1544307

[9] Henzler-Wildman KA, Thai V, Lei M, Ott M, Wolf-Watz M, et al (2007) Intrinsic motions along an enzymatic reaction trajectory. Nature 450: 838–844. 18026086 10.1038/nature06410

[10] McKinney SA, Déclais A–C, Lilley DMJ, Ha T (2003) Structural dynamics of individual Holliday junctions. Nat Struct Biol 10: 93–97. 12496933 10.1038/nsb883

[11] Bokinsky G, Rueda D, Misra VK, Rhodes MM, Gordus A, et al (2003) Single-molecule transition-state analysis of RNA folding. Proc Natl Acad Sci USA 100: 9302–9307. 12869691 10.1073/pnas.1133280100 PMC170913

[12] Rueda D, Bokinsky G, Rhodes MM, Rust MJ, Zhuang X, et al (2004) Single-molecule enzymology of RNA: essential functional groups impact catalysis from a distance. Proc Natl Acad Sci USA 101: 10066–10071. 15218105 10.1073/pnas.0403575101 PMC454165

[13] Zhuang X, Bartley LE, Babcock HP, Russel R, Ha T, et al (2000) A single-molecule study of RNA catalysis and folding. Science 288: 2048–2051. 10856219 10.1126/science.288.5473.2048

[14] Zhuang X, Kim H, Pereira MJB, Babcock HP, Walter NG, et al (2002) Correlating structural dynamics and function in single ribozyme molecules. Science 296: 1473–1476. 12029135 10.1126/science.1069013

[15] Blanco M, Walter NG (2010) Analysis of complex single-molecule FRET time trajectories. Method Enzymol 475: 153–178. 10.1016/S0076-6879(10)72011-5 PMC3012381 20580964

[16] Chung S-H, Kennedy RA (1991) Forward-backward non-linear filtering technique for extracting small biological signals from noise. J Neurosci Meth 40: 71–86. 10.1016/0165-0270(91)90118-j 1795554

[17] Haran G (2004) Noise reduction in single-molecule fluorescence trajectories of folding proteins. Chem Phys 307: 137–145.

[18] Taylor JN, Makarov DE, Landes CF (2010) Denoising single-molecule FRET trajectories with wavelets and Bayesian inference. Biophys J 98: 164–173. 20074517 10.1016/j.bpj.2009.09.047 PMC2800976

[19] Taylor JN, Landes CF (2011) Improved resolution of complex single-molecule FRET system via wavelet shrinkage. J Phys Chem B 115: 1105–1114. 21214275 10.1021/jp1050707

[20] Haar A (1910) Zur Theorie der orthogonalen Funktionensysteme. Math Ann 69: 331–371.

[21] Little MA, Jones NS (2011) Generalized methods and solvers for noise removal from piecewise constant signal. Proc R Soc A 467: 3088–3114. 22003312 10.1098/rspa.2010.0671 PMC3191861

[22] Lee H-C, Lin B-L, Chang W-H, Tu I-P (2012) Toward automated denoising of single molecular Förster resonance energy data. J Biomed Opt 17: 011007. 22352641 10.1117/1.JBO.17.1.011007

[23] Yang H, Xie XS (2002) Statistical approaches for probing single-molecule dynamics photon-by-photon. Chem Phys 284: 423–437.

[24] Yang H, Luo G, Karnchanaphanurach P, Mouie T-M, Rech I, et al (2003) Protein conformational dynamics probed by single-molecule electron transfer. Science 302: 262–266. 14551431 10.1126/science.1086911

[25] Bronson JE, Fei J, Hofman JM, Gonzalez RL Jr, Wiggins CH (2009) Learning rates and states from biophysical time series: a Bayesian approach to model selection and single-molecule FRET data. Biophys J 97: 3196–3205. 20006957 10.1016/j.bpj.2009.09.031 PMC2793368

[26] Greenfield M, Pavlichin DS, Mabuchi H, Herschlag D (2012) Single molecule analysis research tool (SMART): an integrated approach for analyzing single molecule data. PloS ONE 7: e30024. 22363412 10.1371/journal.pone.0030024 PMC3282690

[27] Kelly D, Dillingham MS, Hudson A, Wiesner K (2012) A new method for inferring hidden Markov models from noisy time sequences. PLoS ONE 7: e29703. 22247783 10.1371/journal.pone.0029703 PMC3256161

[28] Qin F, Li L (2004) Model-based fitting of single-channel dwell-time distributions. Biophys J 87: 1657–1671. 15345545 10.1529/biophysj.103.037531 PMC1304571

[29] Eddy SR (2004) What is a hidden Markov model? Nat Biotechnol 22: 1315–1316. 15470472 10.1038/nbt1004-1315

[30] Jung S, Dickson RM (2009) Hidden Markov analysis of short single molecule intensity trajectories. J Phys Chem B 113: 13886–13890. 19785407 10.1021/jp907019p PMC2762486

[31] Lee T-H (2009) Extracting kinetics information from single-molecule fluorescence resonance energy transfer data using hidden Markov models. J Phys Chem B 113: 11535–11542. 19630372 10.1021/jp903831z PMC8785102

[32] Liu Y, Park J, Dahmen KA, Chemla YR, Ha T (2010) A comparative study of multivariate and univariate hidden Markov modelings in time-binned single-molecule FRET data analysis. J Phys Chem B 114: 5386–5403. 20361785 10.1021/jp9057669

[33] König SLB, Kowerko D, Sigel RKO (2013) Kinetic subpopulations detected by single-molecule spectroscopy: fundamental property of functional nucleic acids or experimental artefact? CHIMIA 67: 240–243. 23967697 10.2533/chimia.2013.240

[34] Steiner M, Karunatilaka KS, Sigel RKO, Rueda D (2008) Single-molecule studies of group II intron ribozymes. Proc Natl Acad Sci USA 105: 13853–13858. 18772388 10.1073/pnas.0804034105 PMC2544543

[35] Steiner M, Rueda D, Sigel RKO (2009) Ca^2+^ induces the formation of two distinct subpopulations of group II intron molecules. Angew Chem Int Ed 48: 9739–9742. 10.1002/anie.200903809 PMC2864518 19924747

[36] Antonik M, Felekyan S, Gaiduk A, Seidel CAM (2006) Separating structural heterogeneities from stochastic variations in fluorescence resonance energy transfer distribution via photon distribution analysis. J Phys Chem B 110: 6970–6978. 16571010 10.1021/jp057257+

[37] Gopich IV, Szabo A (2007) Single-molecule FRET with diffusion and conformational dynamics. J Phys Chem B 111: 12925–12932. 17929964 10.1021/jp075255e

[38] Nir E, Michalet X, Hamadani KM, Laurence TA, Neuhauser D, et al (2006) Shot-noise limited single-molecule FRET histograms: comparison between theory and experiments. J Phys Chem B 110: 22103–22124. 17078646 10.1021/jp063483n PMC3085016

[39] Chung HS, Louis JM, Eaton WA (2010) Distinguishing between protein dynamics and dye photophysics in single-molecule FRET experiments. Biophys J 98: 696–706. 20159166 10.1016/j.bpj.2009.12.4322 PMC2820649

[40] Gopich IV, Szabo A (2006) Theory of the statistics of kinetic transitions with application to single-molecule enzyme catalysis. J Chem Phys 124: 154712. 16674256 10.1063/1.2180770

[41] Kalinin S, Sisamakis E, Magennis SW, Felekyan S, Seidel CAM (2010) On the origin of broadening of single-molecule FRET efficiency distributions beyond shot noise limits. J Phys Chem B 114: 6197–6206. 20397670 10.1021/jp100025v

[42] Ha T, Tinnefeld P (2012) Photophysics of fluorescent probes for single-molecule biophysics and super-resolution imaging. Annu Rev Phys Chem 63: 595–617. 22404588 10.1146/annurev-physchem-032210-103340 PMC3736144

[43] Zhao R, Rueda D (2013) Memory effects in RNA folding dynamics revealed by single molecule fluorescence. In: Russell R, editor. Biophysics of RNA Folding (Biophysics for the Life Sciences). New York, NY: Springer. 117–133.

[44] Efron B (1979) Bootstrap methods: another look at the jackknife. Ann Stat 7: 1–26.

[45] Pattengale ND, Alipour M, Bininda-Emonds ORP, Moret BME, Stamatakis A (2010) How many bootstrap replicates are necessary? J Comput Biol 17: 337–354. 20377449 10.1089/cmb.2009.0179

[46] Pla L (2004) Bootstrap confidence intervals for the Shannon biodiversity index: a simulation study. J Agric Biol Envir S 9: 42–56.

[47] Szoszkiewicz R, Ainavarapu SRK, Wiita AP, Perez-Jiminez R, Sanchez-Ruiz JM, et al (2008) Dwell time analysis of a single-molecule mechanochemical reaction. Langmuir 24: 1356–1364. 17999545 10.1021/la702368b

[48] Cheng W, Arunajadai SG, Moffit JR, Tinoco IJ, Bustamante C (2011) Single-base pair unwinding and asynchronous RNA release by the hepatitis C virus NS3 helicase. Science 333: 1746–1749. 21940894 10.1126/science.1206023 PMC4172460

[49] Hoefling M, Lima N, Haenni D, Seidel CAM, Schuler B, et al (2011) Structural heterogeneity and quantitative FRET efficiency distributions of polyprolines through a hybrid atomistic simulation and Monte Carlo approach. PloS ONE 6: e19791. 21629703 10.1371/journal.pone.0019791 PMC3101224

[50] Manly BFJ (2007) Randomization, bootstrap and Monte Carlo methods in biology; Carlin BP, Chatfield C, Tanner M, Zidek J, editors. Boca Raton, FL: Chapman & Hall/CRC.

[51] Kruschel D, Sigel RKO (2008) Divalent metal ions promote the formation of the 5′-splice site recognition complex in a self-splicing group II intron. J Inorg Biochem 102: 2147–2154. 18842303 10.1016/j.jinorgbio.2008.08.006

[52] Pyle AM (2010) The tertiary structure of group II introns: implications for biological function and evolution. Crit Rev Biochem Mol Biol 45: 215–232. 20446804 10.3109/10409231003796523 PMC4408542

[53] Kruschel D, Skilandat M, Sigel RKO (2013) NMR structure of the 5'-splice site in the group IIB intron Sc.ai5γ – conformational requirements for positioning of the exon-intron junction. RNA, accepted. 10.1261/rna.041137.113 PMC3923125 24448450

[54] Walter NG (2001) Structural dynamics of catalytic RNA highlighted by fluorescence resonance energy transfer. Methods 25: 19–30. 11558994 10.1006/meth.2001.1212

[55] Cardo L, Karunatilaka KS, Rueda D, Sigel RKO (2012) Single molecule FRET characterization of large ribozyme folding. Methods Mol Biol 848: 227–251. 22315073 10.1007/978-1-61779-545-9_15

[56] Zhao R, Rueda D (2009) RNA folding dynamics by single-molecule fluorescence resonance energy transfer. Methods 49: 112–117. 19409995 10.1016/j.ymeth.2009.04.017

[57] Selvin PR, Ha T (2007) Single-molecule techniques - a laboratory manual. Cold Spring Harbor, New York, USA: Cold Spring Harbor Laboratory Press.

[58] Kapanidis AN, Lee NK, Laurence TA, Doose S, Margeat E, et al (2004) Fluorescence-aided molecule sorting: analysis of structure and interactions by alternating-laser excitation of single molecules. Proc Natl Acad Sci USA 101: 8936–8941. 15175430 10.1073/pnas.0401690101 PMC428450

[59] Solomatin SV, Greenfield M, Chu S, Herschlag D (2010) Multiple native states reveal persistent ruggedness of an RNA folding landscape. Nature 463: 681–684. 20130651 10.1038/nature08717 PMC2818749

[60] Okamoto K, Sako Y (2012) Variational analysis of a photon-based hidden Markov model for single-molecule FRET trajectories. Biophys J 103: 1315–1324. 22995504 10.1016/j.bpj.2012.07.047 PMC3446682

[61] MacQueen JT (1967) Some observations concerning the van't Hoff equation. J Chem Educ 44: 755–756.

[62] Qin F (2004) Restoration of single-channel currents using the segmental k-means method based on hidden Markov modeling. Biophys J 86: 1488–1501. 14990476 10.1016/S0006-3495(04)74217-4 PMC1303984

[63] Ditzler MA, Rueda D, Mo J, Håkansson K, Walter NG (2008) A rugged free energy landscape separates multiple functional RNA folds throughout denaturation. Nucleic Acids Res 36: 7088–7099. 18988629 10.1093/nar/gkn871 PMC2602785

[64] Schlegel G, Bohnenberger J, Potapova I, Mews A (2002) Fluorescence decay time of single semiconductor nanocrystals. Phys Rev Lett 88: 137401. 11955124 10.1103/PhysRevLett.88.137401

[65] Politis DN (2003) The impact of bootstrap methods on time series analysis. Statist Sci 18: 219–230.

[66] Quinn GP, Keough MJ (2011) Experimental design and data analysis for biologists. Cambridge, United Kingdom: Cambridge University Press.

[67] Su LJ, Qin PZ, Michels WJ, Pyle AM (2001) Guiding ribozyme cleavage through motif recognition: the mechanism of cleavage site selection by a group II intron ribozyme. J Mol Biol 306: 655–668. 11243778 10.1006/jmbi.2000.4323

[68] Kim H-K, Rasnik I, Liu J, Ha T, Lu Y (2007) Dissecting metal ion-dependent folding and catalysis of a single DNAzyme. Nat Chem Biol 3: 763–768. 17965708 10.1038/nchembio.2007.45 PMC4376948

[69] König SLB, Kowerko D, Khier M, Hadzic M, Sigel RKO (2013) Cation-dependent formation of RNA structure dissected by single-molecule fluorescence. Submitted.

[70] Kowerko D, König SLB, Skilandat M, Kruschel D, Cardo L, et al.. (2013) Metal ion induced kinetic heterogeneity of the intron-exon recognition in single group II Intron ribozymes. Submitted. 10.1073/pnas.1322759112 PMC4371910 25737541

[71] Ennifar E, Walter P, Dumas P (2003) A crystallographic study of the binding of 13 metal ions to two related RNA duplexes. Nucleic Acids Res 31: 2671–2682. 12736317 10.1093/nar/gkg350 PMC156032

[72] Hess ST, Girirajan TPK, Mason MD (2006) Ultra-high resolution imaging by fluorescence photoactivation localization microscopy. Biophys J 91: 4258–4272. 16980368 10.1529/biophysj.106.091116 PMC1635685

[73] Kapanidis AN, Laurence TA, Lee NK, Margeat E, Kong X, et al (2005) Alternating-laser excitation of single molecules. Acc Chem Res 38: 523–533. 16028886 10.1021/ar0401348

[74] Giudici P, Rydén T, Vandekerkhove P (2000) Likelihood-ratio tests for hidden Markov models. Biometrics 56: 742–747. 10985210 10.1111/j.0006-341x.2000.00742.x

[75] Krause S, Kowerko D, Börner R, Hübner CG, von Borczyskowski C (2011) Spectral diffusion of single molecules in a hierarchical energy landscape. ChemPhysChem 12: 303–312. 21275022 10.1002/cphc.201000678

[76] Börner R, Kowerko D, Krause S, Von Borczyskowski C, Hübner CG (2012) Efficient simultaneous fluorescence orientation, spectrum, and lifetime detection for single molecule dynamics. J Chem Phys 137: 164202. 23126703 10.1063/1.4759108

[77] Hellriegel C, Kirstein J, Bräuchle C, Latour V, Pigot T, et al (2004) Diffusion of single streptocyanine molecules in the nanoporous network of sol-gel glasses. J Phys Chem B 108: 14699–14709.

[78] Lee T-H, Lapidus LJ, Zhao W, Travers KJ, Herschlag D, et al (2007) Measuring the folding transition time of single RNA molecules. Biophys J 92: 3275–3283. 17307831 10.1529/biophysj.106.094623 PMC1852359

[79] Okamoto K, Sannohe Y, Mashimo T, Sugiyama H, Terazima M (2008) G-quadruplex structures of human telomere DNA examined by single-molecule FRET and BrG-substitution. Bioorgan Med Chem 16: 6873–6879. 10.1016/j.bmc.2008.05.053 18555689

[80] Okamoto K, Terazima M (2008) Distribution analysis for single-molecule FRET measurement. J Phys Chem B 112: 7308–7314. 18491936 10.1021/jp712104h

