# Supplementary figures and images for: BOBA FRET: Bootstrap-Based Analysis of Single-Molecule FRET Data
Source: PLoS One. 2013 Dec 27;8(12):e84157. doi: 10.1371/journal.pone.0084157 (PMC3873958; doi:10.1371/journal.pone.0084157)

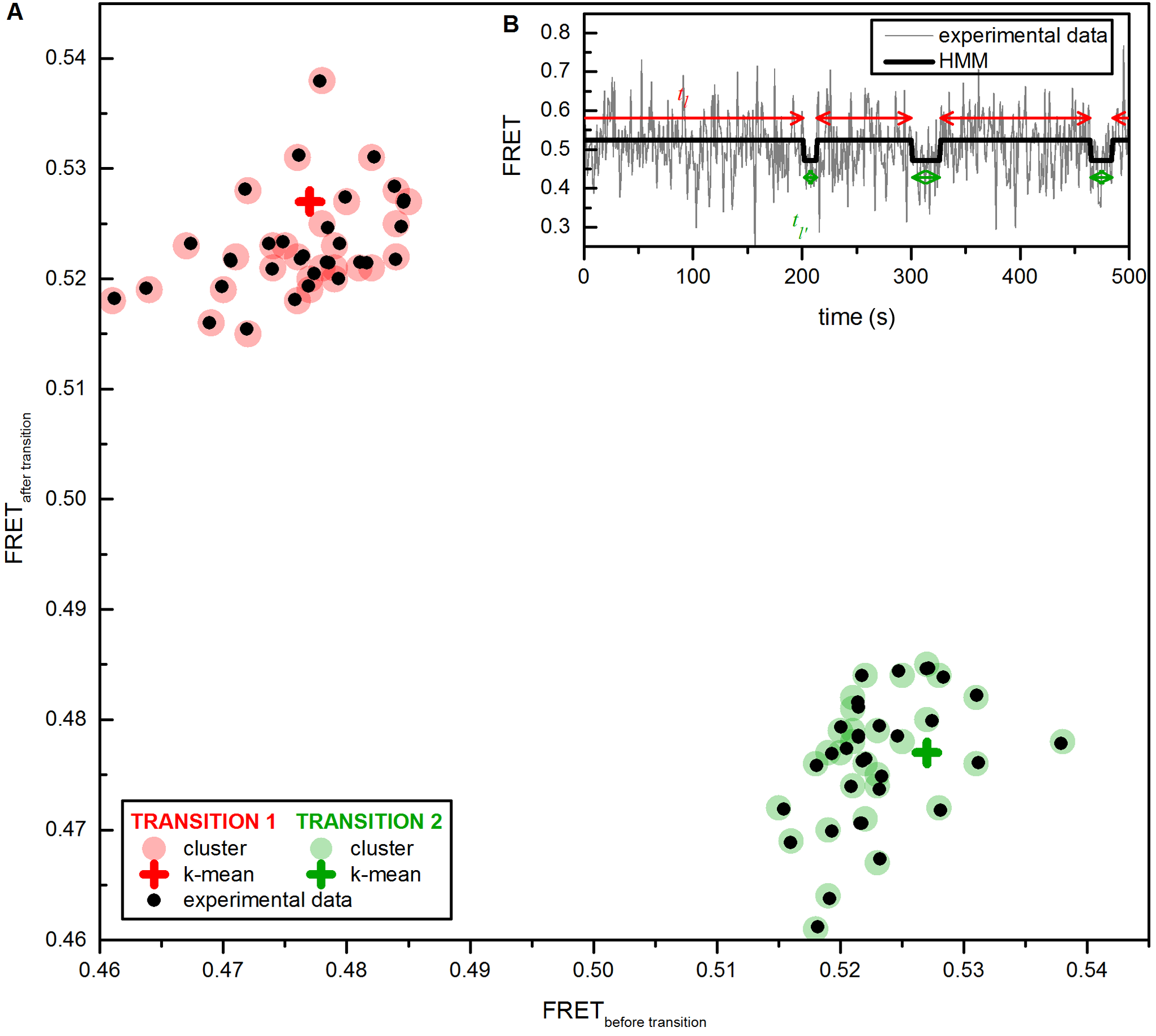

Supplement: Figure S1 — k-means clustering to assign dwell times to consistent FRET values for further processing steps. (A) Transition density plot (TDP) built from a set of HMM-discretized FRET time traces. The data points are iteratively assigned to one of the two centers according to their distance. The center coordinates are then recalculated according to the distances and occurrences (weights) of the clustered data point. The weighted k-mean centers are assumed to be definitive when the set of clustered transition does not change after an additional round of iteration. (B) Dwell time analysis of one simulated FRET time trace for a two state system: ΔFRET = 0.04, FRET A = 0.48 (undocked state), FRET B = 0.52 (docked state); SNR = 6.0 (width σ = 0.143); observation time = 4000 s (magnified to highlight transitions); k docking = 0.04 s–1 (intramolecular reaction) k undocking = 0.1 s–1. Each of the two FRET states detected in the trace are assigned to the center of one of the two clusters and the corresponding dwell times are subsequently used for thermodynamic or kinetic analysis. (TIF) [file pone.0084157.s001.tif]

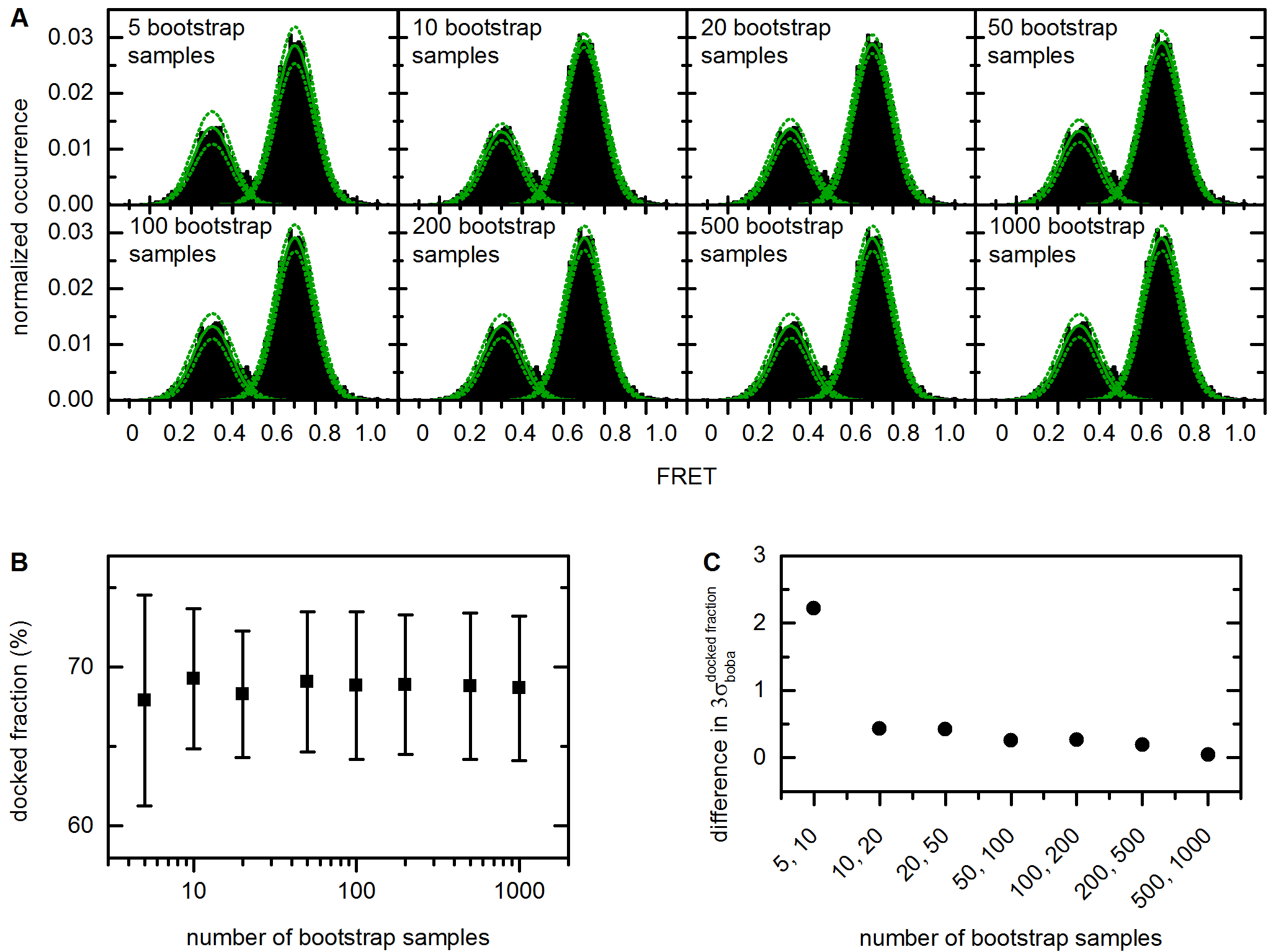

Supplement: Figure S2 — Dependence of the bootstrapped estimated cross-sample variability on the number of bootstrap samples. (A) Gaussian fitting was performed in conjunction with bootstrapping to analyze 100 simulated smFRET time traces (N = 100, Eq. (8)). The number of bootstrap samples was varied between 5 and 1000 (M, Eq. (9)). The histogram corresponds to the normalized cumulated histogram built from all time traces (Eq. (3)), solid lines depict Gaussian fit functions, dashed lines the variability associated with the amplitude and the width (3σ boba). (B) Fraction of docked molecules and cross-sample variability, data from panel (A). Error bars correspond to 3*σ boba. (C) Dependence of Δσ boba on the number of bootstrap samples. Data point correspond to the difference in 3*σ boba of adjacent data points and demonstrate that fluctuations become negligible when more than 100 bootstrap samples are used. Parameters of the simulation: FRET A = 0.3 (undocked state), FRET B = 0.7 (docked state); SNR = 3.5; observation time = 100 s; k docking = 0.1 s–1, k undocking = 0.04 s–1. (TIF) [file pone.0084157.s002.tif]

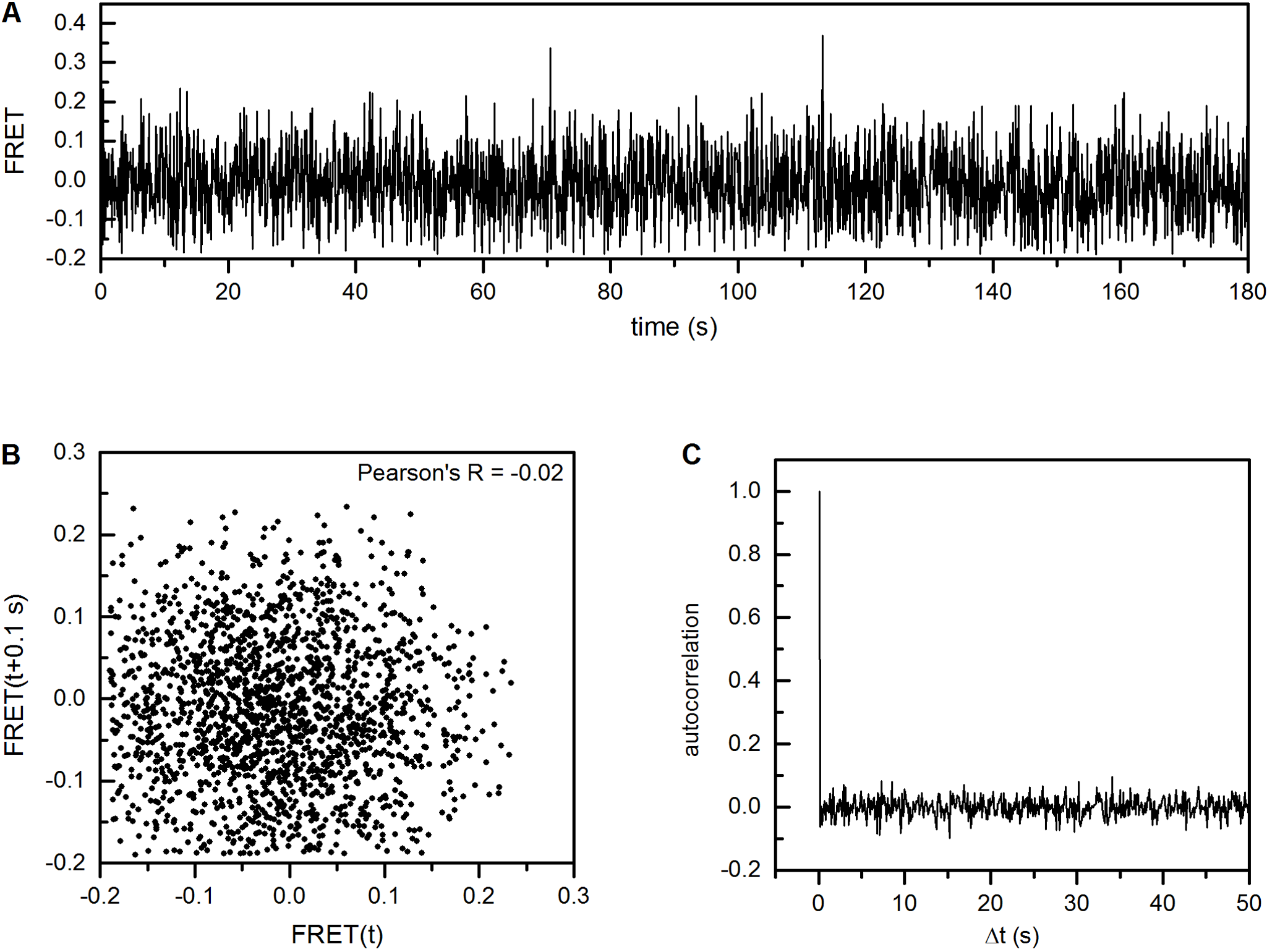

Supplement: Figure S3 — Statistical nature of noise in smFRET data. (A) Cy3 emission time trace, representative data. Surface-tethered Cy3-tagged d3'EBS1* fluctuates around zero FRET in the absence of IBS1*. (B) FRET(t) versus FRET(t +100 ms) scatter plot of the data shown in (A) develops as a two-dimensional Gaussian distribution. Time-dependent noise would be expected to accumulate on a diagonal. (C) The autocorrelation function of the data in (A) clearly demonstrates that the noise of the time trace shown in (A) is time-independent. (TIF) [file pone.0084157.s003.tif]

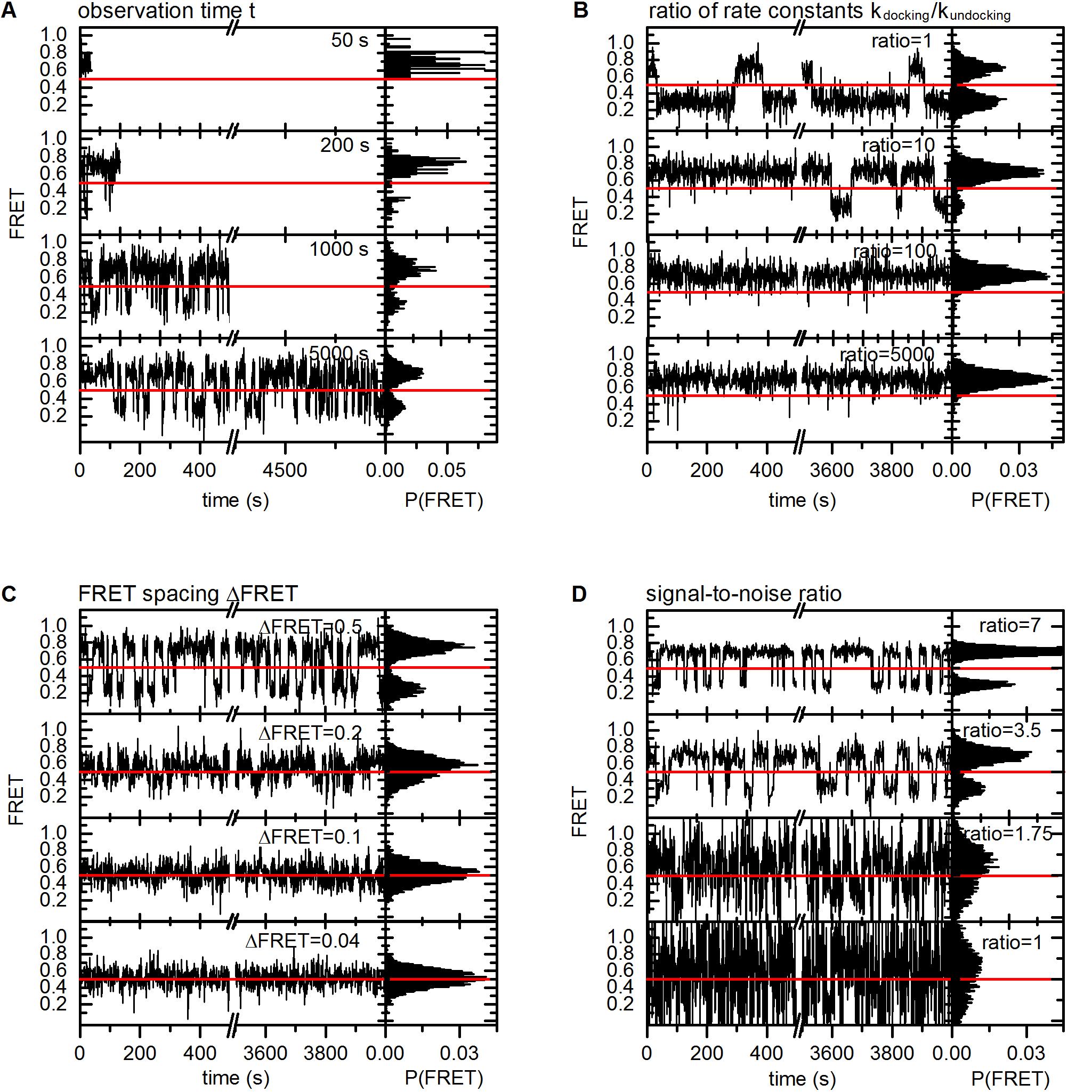

Supplement: Figure S4 — Representative data of simulated smFRET time traces and normalized histograms, representative data. Standard parameters of the simulation: FRET A = 0.3 (undocked state), FRET B = 0.7 (docked state); SNR = 3.5; observation time = 4000 s; k docking = 0.1 s–1, k undocking = 0.04 s–1. (A) The observation time is varied between 50 s and 4000 s (1 frame per second). (B) The ratio of rate constants associated with “docking” and “undocking” is changed from 1 to 5000 (k undocking = 0.005 s–1 = constant; 0.005 s–1≤ k docking ≤25 s–1). (C) The spacing of the centers of the FRET distributions is varied from 0.5 to 0.02. (D) The signal-to-noise ratio is varied from 7 to 1. (TIF) [file pone.0084157.s004.tif]

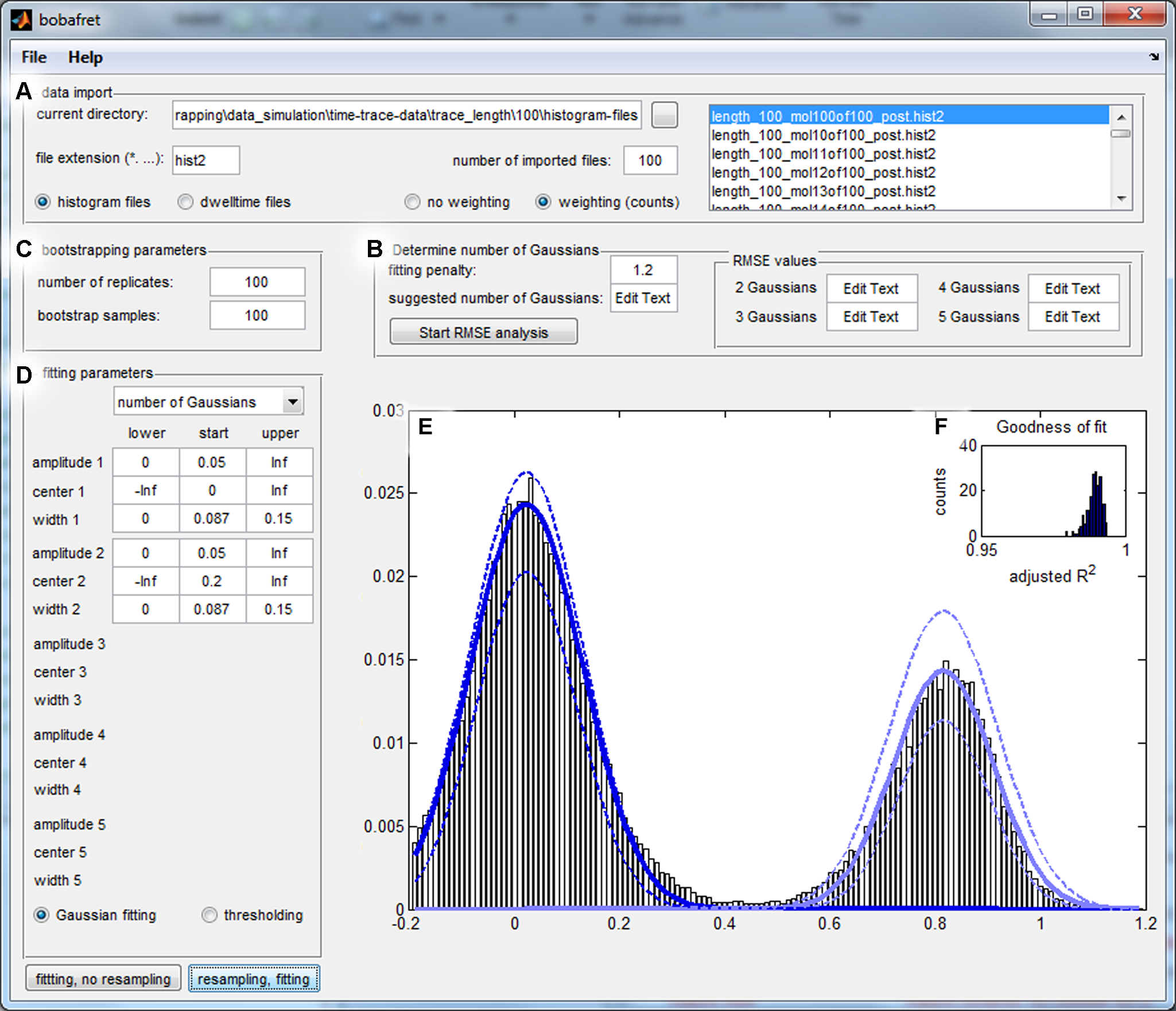

Supplement: Figure S5 — Boba FRET user interface for thermodynamic analysis. (A) Data import from ASCII files. Both smFRET histogram files (first column: FRET, second column: occurrence (counts); further columns are ignored) and dwell time files are supported (first column: duration, second column: FRET before transition, third column: FRET after transition). (B) Optional determination of the optimal number of Gaussians by distribution analysis [79], [80]. (C) Setting the parameters for bootstrapping (N and M, Eqs. (7) and (8) in the main text). (D) Setting the starting guesses and boundaries of the Gaussian fits (Eq. (13) in the main text). Alternatively, thresholding can be performed. (E) Original normalized data and fitting results. Solid lines correspond to the fit to the original data, dashed lines to the bootstrapped estimated variability (highest and lowest values of the amplitude and the width). (F) Goodness of fit to all bootstrapped histograms. All fitting parameters (in the case of Gaussian fitting) and the relative occurrences are automatically exported to text files for further analysis. (TIF) [file pone.0084157.s005.tif]

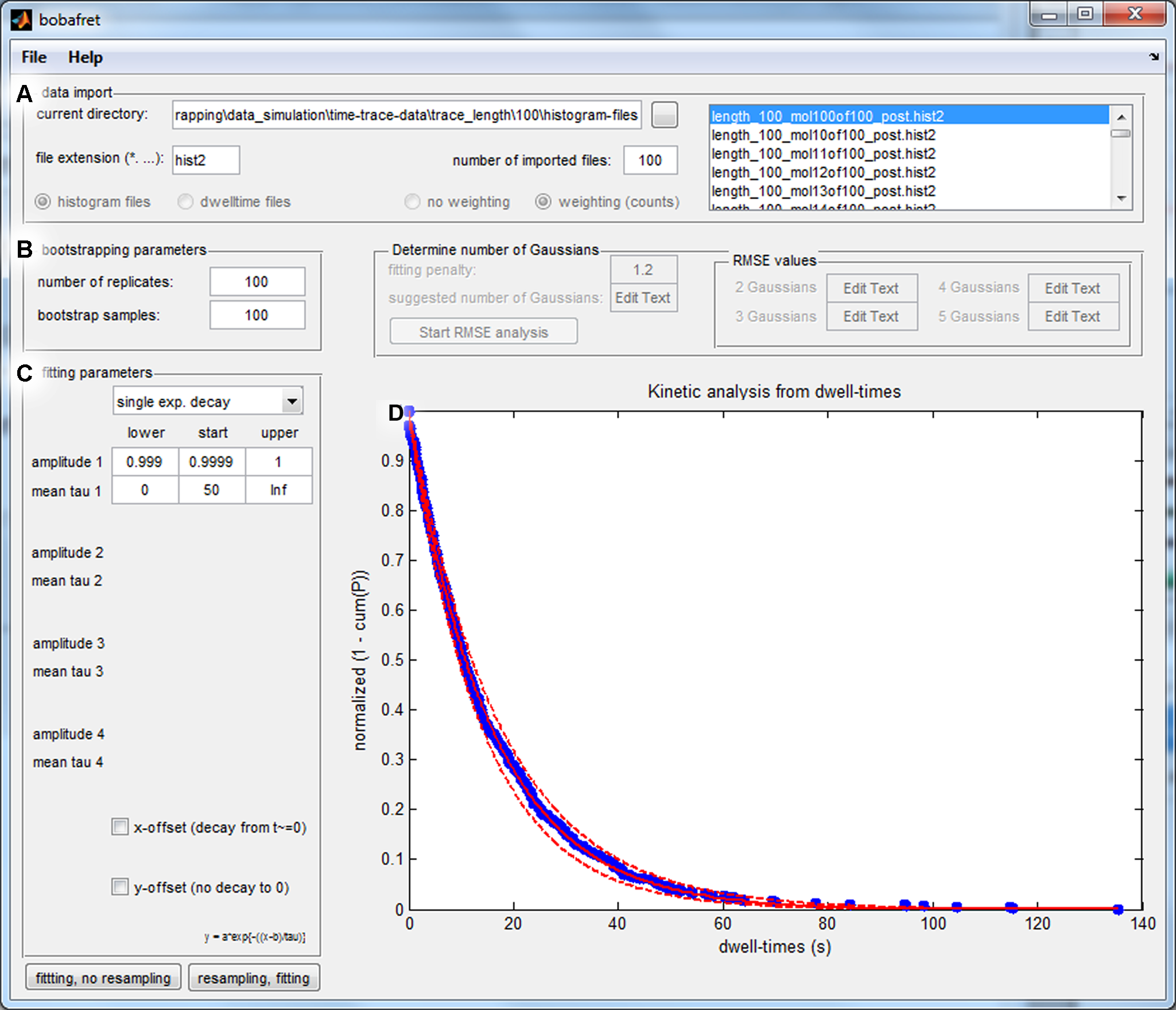

Supplement: Figure S6 — Boba FRET user interface for dwell time analysis. (A) Data import from ASCII files. File format: first column, duration; second column, FRET before transition; third column, FRET after transition. (B) Setting the parameters for bootstrapping (N and M, Eqs. (7) and (8) in the main text). (C) Setting the starting values and boundaries of the exponential decay function to be used for fitting. Mono-, bi-, tri-, and tetraexponential decays functions are implemented, as well as stretched exponential decays (Eqs. (3) and (4) in the main text). (D) Original normalized data and fitting results. Solid lines correspond to the fit to the original data, dashed lines to the bootstrapped estimated variability (highest and lowest values for the decay constant). All fitting parameters are automatically exported to text files for further analysis. (TIF) [file pone.0084157.s006.tif]
